# The Growing Interest in Development of Innovative Optical Aptasensors for the Detection of Antimicrobial Residues in Food Products

**DOI:** 10.3390/bios10030021

**Published:** 2020-03-03

**Authors:** Valérie Gaudin

**Affiliations:** Project Manager in Biochemistry, Anses, Laboratory of Fougeres, European Union Reference Laboratory (EU-RL) for Antimicrobial and Dye Residue Control in Food-Producing Animals, Bâtiment Bioagropolis—La Haute Marche-Javené, 35302 Fougères, France; valerie.gaudin@anses.fr; Tel.: +33-2-99-17-27-47

**Keywords:** aptasensors, screening, antimicrobial residues, food products, optical, nanomaterials, multiplexing

## Abstract

The presence of antimicrobial residues in food-producing animals can lead to harmful effects on the consumer (e.g., allergies, antimicrobial resistance, toxicological effects) and cause issues in food transformation (i.e., cheese, yogurts production). Therefore, to control antimicrobial residues in food products of animal origin, screening methods are of utmost importance. Microbiological and immunological methods (e.g., ELISA, dipsticks) are conventional screening methods. Biosensors are an innovative solution for the development of more performant screening methods. Among the different kinds of biosensing elements (e.g., antibodies, aptamers, molecularly imprinted polymers (MIP), enzymes), aptamers for targeting antimicrobial residues are in continuous development since 2000. Therefore, this review has highlighted recent advances in the development of aptasensors, which present multiple advantages over immunosensors. Most of the aptasensors described in the literature for the detection of antimicrobial residues in animal-derived food products are either optical or electrochemical sensors. In this review, I have focused on optical aptasensors and showed how nanotechnologies (nanomaterials, micro/nanofluidics, and signal amplification techniques) largely contribute to the improvement of their performance (sensitivity, specificity, miniaturization, portability). Finally, I have explored different techniques to develop multiplex screening methods. Multiplex screening methods are necessary for the wide spectrum detection of antimicrobials authorized for animal treatment (i.e., having maximum residue limits).

## 1. Introduction

### 1.1. Antimicrobial Residues in Food Products

Antimicrobials are administered to farm animals for curative purposes. Therefore, antimicrobial residues could be found in animal food products (e.g., milk, meat, eggs, honey, fish), depending on the pharmacokinetic properties of the veterinary medicines. Some antimicrobial residues are allowed to be present, but below a fixed concentration, called the maximum residue limit (MRL), which is set in the European regulation [1,2]. These levels were established because the presence of antimicrobial residues could lead to potential consumer health risks (e.g., allergies, toxicological risks, antimicrobial resistance) and could cause issues in the transformation of food products (e.g., the transformation of milk into cheese, yogurts). Antimicrobials can be classified on the basis of their chemical structure. Each family of antimicrobial is characterized by a common chemical structure (e.g., beta-lactam ring for beta-lactams). Antimicrobials within a family can vary at the level of their side chain or with the addition of cycles. The main families of antimicrobials to be detected in animal-derived food products are beta-lactams, tetracyclines, macrolides, aminoglycosides, quinolones, and sulfonamides. Each family is constituted with a variable number of substances that could vary from four substances (tetracyclines) to more than 25 (sulfonamides). More than 80 different antimicrobial substances have MRL in animal-derived food products.

Some other antimicrobial residues are banned for livestock animals, due to toxicological risks (e.g., nephrotoxicity, genotoxicity). These substances have to be detected at as low concentrations as possible, which are often below regulatory limits called reference point for action (RPA) [2,3].

According to the European Union regulation [4], all the member states organize national residue monitoring plans (NRMP) for the control of antimicrobial residues in animal-derived food products. The first step of the control is performed with screening methods. Each European Union member state is free to use the screening method of their choice, providing that the method has been validated according to the EU regulation [5].

### 1.2. Screening Methods

For banned substances (e.g., chloramphenicol (CAP)), the levels of detection have to be very low because antimicrobial residues have to be detected in food matrices at concentrations (i.e., RPA) ranging from 0.1 to 1 ng/mL (or ng/g). Moreover, the method has to be very specific for each compound to be certain about the presence of illicit antimicrobial and to prevent the occurrence of false-positive results. For MRL substances, the levels of detection should be below MRL, which is generally ranging from 4 to 1500 ng/mL (or ng/g), depending on the antimicrobial family and food matrices. For practicality, screening methods for MRL substances should be selective for at least one family of antimicrobials (e.g., tetracyclines) and not only for one analyte (e.g., oxytetracycline). A wide spectrum of detection is a pre-requisite for a screening method for MRL substances. Therefore, multiplex methods should be developed. The multiplex detection can consist of detecting simultaneously whole family or several families of antimicrobials. Even if the screening method is not able to identify the antimicrobial, further screening methods (e.g., LC-MS/MS) can be used to define the precise nature of the molecule.

Methods based on the inhibition of bacterial growth (e.g., plate tests, tube tests) are conventional methods for screening antimicrobial residues [6,7]. These methods have a wide spectrum of detection that is important for a screening method for MRL substances and are cheap. However, these methods often lack sensitivity for some antimicrobials. Immunological methods like ELISA tests [8,9] and dipsticks [10,11] are implemented for more specific detection of one or several classes of antimicrobial residues or for banned substances. Their sensitivity is sometimes limited, especially since the RPAs are lower [3]. Physico-chemical methods (e.g., HPLC, LC-MS/MS) can be used as screening methods. They are able to identify the analyte and to detect antimicrobial residues below regulatory limits (e.g., MRL, RPA). However, LC-MS/MS methods require expensive equipment, skilled people, and they are time and money consuming [12,13]. Despite these, monitoring plans for antimicrobial residues in Europe are gradually moving towards the use of LC-MS/MS screening, especially because the evaluation of human exposure to residues, even under the MRLs, is now requested.

Therefore, there is a need to develop methods capable of competing with LC-MS/MS (sensitivity, selectivity/specificity) but much cheaper for the official control of antimicrobial residues in food products. In addition to official control, self-monitoring in the industry (e.g., dairies) or field control (e.g., farm, slaughterhouses) requires inexpensive, quick, portable, and easy to set up screening methods. Biosensors have the potential to meet these expectations and compete with current methods at a better cost.

### 1.3. Biosensors

Biosensors are innovative methods that can be used in the field of antimicrobial residue detection. They have been developed for dozens of years for clinical and forensic applications [14,15,16,17]. Food safety applications of biosensors that are more recent could benefit from these developments. A biosensor is a combination of a biosensing element with a transducer that transforms biological reactions into a readable signal [18].

Transducers can be classified into four categories: optical, electrochemical, mass sensitive, and thermal, and have been reviewed elsewhere [19]. Most of the aptasensors in the literature for the detection of antimicrobial residues in animal-derived food products are either optical or electrochemical sensors. Only optical aptasensors are highlighted in this review.

**Figure 1:**
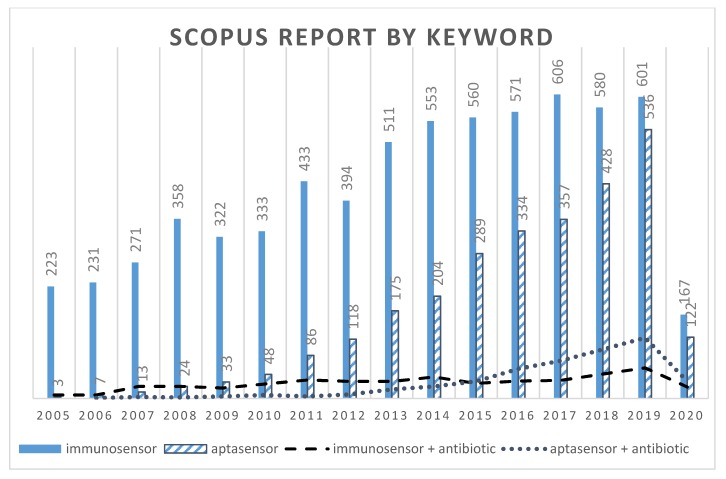
Results of a keyword search on Scopus: numbers of articles published from 2005 to 2020 regarding “immunosensor”, “aptasensor”, “immunosensor AND antibiotic”, and “aptasensor AND antibiotic”.

I have previously reviewed the different biosensing elements used for the detection of antimicrobial compounds (e.g., antibodies, enzymes, cells, molecularly imprinted polymers (MIPs), aptamers) [19]. Their advantages and drawbacks have already been discussed in this review. Polyclonal and monoclonal antibodies are currently used to develop analytical methods based on biomolecular recognition of a target analyte. I have observed that immunosensors were first developed, based on the detection of antimicrobial compounds by antibodies, probably because antibodies were already available for ELISA tests [20,21,22,23]. However, usually, antimicrobials are not immunogenic. They have to be bound to proteins (e.g., bovine serum albumin (BSA)) to produce antibodies. More recently, innovative biosensing elements like MIPs, and especially aptamers, are in growing development. The use of aptamers as a very promising alternative to antibodies to develop biosensors was already reported in 2005 [24]. Since that date, the volume of articles in the literature dealing with aptasensors is increasing regularly, while immunosensors are stable (Figure 1).

Therefore, I have focused this review on optical aptasensors to develop detection tools for antimicrobial residues in animal-derived food products. Fluorescent and colorimetric aptasensors have been highlighted in this review because they are the most developed optical aptasensors. Advances in nanotechnologies (nanomaterials, micro/nanofluidics, and signal amplification techniques) that largely contribute to improved performance (sensitivity, specificity, miniaturization, portability) of aptasensors have been presented. Finally, multiplex screening methods bring benefits of wide spectrum detection of authorized antimicrobials. Different techniques to develop multiplex methods are explored in this review.

## 2. Aptamers for Antimicrobial Residues

Aptamers are oligonucleotide sequences (single-stranded DNA or RNA molecules), which recognize and bind to a specific target molecule with a defined affinity and specificity [25].

### 2.1. Aptamer Production

A multi-step iterative process of in vitro selection and amplification cycles that is called SELEX (systematic evolution of ligands by exponential enrichment) produces aptamers [26]. The first step is the selection of the target-binding DNA from a library of oligonucleotides, consisting of randomly created sequences. The second step is the elimination of non-binding DNA (washings) and the elution of the target-binding DNA. The third step is the amplification of the target-binding DNA by polymerase chain reaction (PCR). The repetition of these three steps enriches the target-binding DNA solution.

In the beginning, aptamers were produced to target proteins [27,28,29,30]. Nevertheless, thereafter some aptamers were developed to bind to small molecules, like antimicrobials. Advances in the production of aptamers for small molecules and the development of analytical methods based on aptamers (i.e., aptasensors) were reviewed by Kim et al. [31].

### 2.2. Aptamers’ Characteristics: Advantages and Drawbacks

Aptamers appear for about 20 years as the rival of antibodies (polyclonal, monoclonal) to develop binding assays, and especially for the development of diagnostic tests [32,33,34,35]. Antibodies have been used for the development of analytical methods for dozens of years. They allowed the development of many immunological methods (e.g., ELISA, dipsticks, immunosensors), for the detection of antimicrobial residues in clinical studies and in food products also, with good performance. The development of aptasensors to detect antimicrobial residues is still in its infancy compared to immunosensors.

The production of antibodies is laborious and expensive and could be challenging when the target analyte is not an immunogen. Aptamers are not limited to immunogens, and the target could be small molecules, proteins, metallic ions, etc. The synthesis of aptamers is less expensive than the production of antibodies, simpler, reproducible, and could be automated. However, the first step of the selection of the aptamer by the SELEX method could be difficult and time-consuming.

The performance of assays developed with polyclonal antibodies varies from batch to batch, while the assays developed with monoclonal antibodies are more stable because monoclonal antibodies can be prepared in large and renewable amount, and the supply is reproducible [21,36]. Aptamers are more resistant to hard conditions (e.g., temperature, long-term storage, chemical solvents, and non-physiological buffer). The stability of immobilized aptamers is better than the stability of immobilized antibodies (transport and storage can be done at room temperature). Denatured aptamers could be regenerated easily, which is not the case of antibodies.

Aptamers present some advantages in comparison with antibodies [25]. Aptamers are more flexible than antibodies because their production is not dependent on an animal (polyclonal antibodies) or cells (monoclonal antibodies). Therefore, they can be modified to improve their affinity for the target or their stability. Hasegawa et al. (2016) discussed the impact of aptamer sequences on their structure and their binding affinity [37]. Aptamers with high affinity are not always obtained after the selection. The optimization of aptamer primary sequences is one method that could be used to improve the binding affinity of aptamer towards its target. For instance, a 76-mer ssDNA aptamer has been truncated to produce a shortened aptamer (8-mer ssDNA) that has a much higher affinity for four tetracyclines [38]. Therefore, the sensitivity of the colorimetric is 500-fold enhanced compared to that obtained using the 76-mer aptamer. Furthermore, chemical modification of aptamers can be performed to improve their stability (e.g., stabilization of aptamer conformations) [39].

One major advantage of aptamers is that they are easily modifiable via the addition of functional groups. Aptamers could be immobilized onto a surface through functional groups that can be added during their chemical synthesis (e.g., biotinylated CAP aptamer-functionalized magnetic nanoparticles (MNPs) [40], thiol modified aptamer conjugated on gold nanoparticles (AuNPs) [41]), as well as reporter molecules (e.g., fluorescein, biotin) can be combined with aptamers (e.g., carboxyfluorescein (FAM)-labeled aptamer [42], cyanine 3-labeled aptamer [43], horseradish peroxidase (HRP)-labeled aptamer by biotin-streptavidin system [44]). Aptamers can be labeled without loss of affinity. Aptamers can discriminate minute changes into the structure of a molecule (e.g., methyl group, hydroxyl group) [25]. It is an advantage when high specificity is required (e.g., banned antimicrobial residues). However, it is a strong disadvantage when simultaneous detection of an antibiotic family (multiplexing) is required (e.g., penicillin group). In this last case, the best binding element would be the one that is not able to discriminate between the compounds of a family. One solution to overcome this drawback is to mix several aptamers, each one specific for one target, to recognize the entire family. Therefore, the aptamers offer the possibility of designing a multiplex format for aptasensors [45,46,47,48,49]. The other solution is to produce multifunctional aptamers. Wu et al. (2020) developed a multi-functional aptamer for binding to tetracycline and chloramphenicol [50]. The aptamer contains two recognition fragments with unequal lengths, one short for tetracycline, and one longer for CAP (one-fifth longer).

The affinity of aptamers for small molecules like antimicrobials is often lower than the affinity for proteins. The reason is probably that the surface of contact between the aptamer and the target is lower with small molecules, and so the number of functional groups available for interaction is reduced. Specificity is closely related to affinity. The stronger the affinity, the higher the specificity. Therefore, a minimal change in the chemical structure of the target results in a significant decrease in the interaction with the aptamer because the surface of contact decreases. Aptamers can discriminate targets with subtle structural differences. A strong affinity aptamer-target increases the sensitivity of the developed aptasensor. The interaction between an aptamer and its target analyte can be detected by several mechanisms [51]:

- A change of mass can be detected with mass-sensitive (e.g., quartz crystal microbalance (QCM), surface acoustic wave (SAW)) or optical biosensors (e.g., surface plasmon resonance (SPR));
- A change of its structure and/or conformation (e.g., hairpin, pseudo-node), to fully interact with the target, could be detected by optical or electrochemical biosensors, when, for instance, the 3′ and 5′ extremities of the aptamer are labeled with specific tags (e.g., pairs fluorophores (e.g., carboxyfluorescein (FAM), cyanine)—quenchers (e.g., nanomaterials (e.g., quantum dots (QDs), gold nanoparticles (AuNPs)), or electrochemical tags (methylene blue (MB), ferrocene)). The conformation of a given aptamer is heavily affected by various factors, such as temperature, pH, type, and concentration of cations.
- A change in electrochemical properties could be detected, even without labeling. Aptamers are polyanionic molecules. Therefore, their electrochemical properties could be used for the detection of a target analyte. A change in conductance could be observed and measured when the target analyte is bound to the aptamer compared to when the aptamer is free.

The major drawback of aptamers at the moment is that very few are commercialized, especially for the recognition of antimicrobial residues. There is a combination of issues impeding the widespread application of aptamers in the control of antimicrobial residues in foodstuffs. The reasons for the lack of aptamers available could be both technical and economical. Even though many improvements occurred in the aptamer production since 30 years, the selected aptamers sometimes do not have the desired affinity and specificity [52]. Progresses in SELEX procedures to produce performing aptamers with high binding capacity and specificity are always needed, especially for small molecules. There is a need for standardized kits and protocols based on well-characterized aptamers with optimum characteristics. Furthermore, the aptamers have been developed first for proteins; most of the selection protocols are not adaptable to small molecules like antimicrobials. Specific problems are encountered to produce aptamers for small molecules (e.g., a limited number of functional groups for the interaction target-aptamer and for immobilization during the selection process, aptamer/target affinity difference between immobilized target and target in solution) [53]. For instance, target immobilization before incubation with oligonucleotide libraries for the aptamer selection could modify the structure of the target from its native state. Therefore, the binding affinity of the aptamer to the native state in solution may be not satisfactory. Nevertheless, technical difficulties could not explain completely the lack of aptamers. For many antimicrobials, nobody has tried to produce aptamers. One reason could be that the point of care diagnostics market for the detection of antimicrobials for veterinary applications is probably smaller than the one for human health (e.g., diagnosis of human diseases, foodborne pathogens). Furthermore, antibodies have dominated the market for decades in the field of immunoassays to detect antimicrobial residues in food products (e.g., ELISA, dipsticks). Industries can be reluctant to develop aptamers to replace the antibodies in which they have already invested a lot of money for research and production [54]. For the moment, few aptamer companies have translated their research into viable commercial diagnostic products or commercial reagents. Developers of aptamers for antimicrobial residues should be convinced that they have to invest in aptamers development for diagnostic applications, especially in the field of detection of antimicrobial residues. Some commercial kits now available for other food contaminants could convince industries of the interest to develop aptamer-based diagnostic assays for antimicrobials (e.g., OTA-Sense kit, by Neoventures Biotechnology Inc. (London, Ontario, Canada) for the detection of Ochratoxin A (OTA); AflaSenses kit, by Neoventures Biotechnology Inc. for the detection of aflatoxins).

Nevertheless, it is possible to order custom aptamers on demand from several manufacturers (e.g., Sangon Biotechnology Co. Ltd. (Shanghai, China), Integrated DNA Technologies, Inc. (IDT) (Coralville, Iowa, USA), Neo Ventures Biotechnology (London, Ontario, Canada), Aptamer Sciences Inc. (AptSci, South Korea), Base Pair (Houston, TX, USA)).

### 2.3. Aptamers for Antimicrobials

The first articles about aptamers were published in the 1990s [55], especially for human thrombin binding [27]. The development of aptamers for binding to antimicrobials started to emerge in 2000s (e.g., chloramphenicol (CAP) [56,57], florfenicol [58], malachite green [59], tetracyclines (e.g., oxytetracycline [60], tetracycline [61], tetracyclines (multiplex) [62]), moenomycin [63], penicillin [64]). A review of existing aptamers towards antibiotics (e.g., aminoglycosides, tetracyclines) was published in 2006 [65].

Mehlhorn et al. (2018) provided a comprehensive overview of aptamer sequences already published for the binding to antibiotics and related aptasensors [66]. This review highlighted the fact that most of the aptasensors are focused on a limited number of antibiotics (i.e., aminoglycosides (e.g., kanamycin), chloramphenicol, tetracyclines (tetracycline and oxytetracycline), fluoroquinolones, beta-lactams) compared to a large number of antibiotics to be detected, for instance, in food products. Furthermore, the affinity of the aptamer for its target must be strong, especially when developing aptasensors are based on target-induced strand displacement by complementary DNA (c-DNA). Most of the aptamers are DNA aptamers and not RNA aptamers, even if the affinity of RNA aptamers for their target is stronger than DNA aptamers. The reason is that RNA aptamers degrade more easily and faster.

Analytical methods can be developed based on antibiotic-specific aptamers. The affinity and the selectivity of the aptamer for the target depend on the different sequences of the aptamers. Different sequences can recognize the same target analyte [67] but with different affinities. Some aptamers are very specific (targeted on one antibiotic only) [56], and some others are more selective as they can bind to several antibiotics (multiplex) (e.g., one aptamer recognizes kanamycin, gentamicin, and tobramycin but with a much higher affinity for tobramycin [68]). High specificity is required for banned substances like chloramphenicol, but for authorized substances, it is preferable and cost-effective to detect several antibiotics from the same family with only one aptamer.

## 3. Aptasensors for the Detection of Antimicrobial Residues

Biosensors using an aptamer as a recognition element are called “aptasensors”. According to Piro et al. (2016), the sensitivity of immunosensors seems to be often better than the sensitivity of aptasensors [69]. However, recent progress in transduction and signal amplification has led to the development of many sensitive aptasensors. There is still a long way to cover prior we develop optimal aptasensors for the screening of antimicrobial residues in food products. Indeed, immunoassays are predominant in the market because antibodies are still widely available, even if their specificity and affinity are not always optimal.

Nevertheless, the technology is progressing, and different types of transducers (e.g., optical, electrochemical, mass sensitive, thermal) can be used for the detection of signals from aptasensors. Here, I have described the recognition of antimicrobial residues by aptamers in animal-derived food products, combined with different kind of optical transducers, as well as different techniques of signal amplification. It is noteworthy that the application of nanomaterials in the service of biosensors is growing with the purpose of delivering sensitive aptasensors for the detection of food contaminants [70].

Current optical biosensors are based on different optical principles, such as fluorescence, colorimetry, (chemi) luminescence (CL), surface plasmon resonance (SPR), and surface-enhanced Raman scattering (SERS). 

### 3.1. Fluorescent Aptasensors

The main advantages of optical biosensors, like fluorescence and colorimetric aptasensors, are the relatively simple detection procedures and the minimal usage of analytical instruments. Fluorescent aptasensors are one of the most common optical aptasensors developed over the past ten years for the detection of antimicrobial residues in food products [71]. Fluorescent aptasensors are quick, sensitive, easy to manipulate, and cost-effective. Applications of fluorescent aptasensors to the detection of antimicrobial residues in animal-derived food products are presented in Table 1.

**Figure 2:**
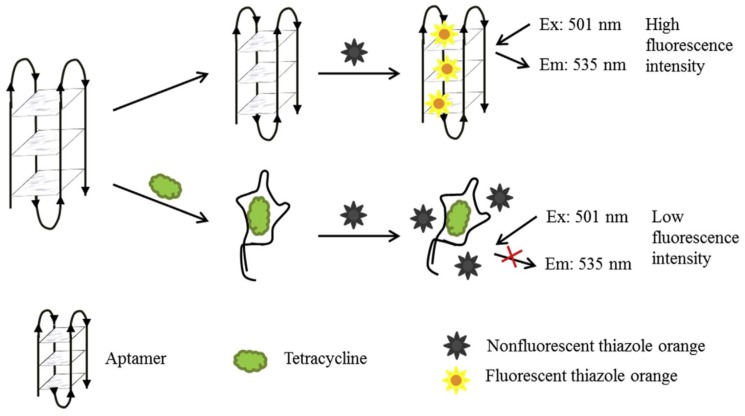
Schematic illustration of the label-free fluorescent probe based on structure-switching aptamers for tetracycline detection [101]. Reprinted from [101]; with permission from Elsevier.

Label-free fluorescence aptasensors can be based on DNA intercalating dyes (e.g., Ru (phen)_2_(dppz))2+, SYBR Green I [96], thiazole orange (TO) [101], metal nanoparticles (NPs) [109]). The detection is based on the fluorescence signal of intercalating dyes (e.g., TO) that is low in solutions, but increases when the dye intercalates into dsDNA (Figure 2). When the target is absent, the dye TO intercalates into the aptamer structure, and the emitted fluorescence is strong. When the target is present, the aptamer binds to the target, and so the intercalating dye is released into the solution, and the fluorescence decreases.

New fluorescent probe curcumin has been tested to improve the sensitivity of fluorescent aptasensors compared to classical synthetic dyes (e.g., SYBR Green I) that bind strongly to the aptamer sequence and result in low detection limits [110]. The aptasensor based on curcumin dye was at least six orders of magnitude more sensitive than sensor constructed with the synthetic dye SYBR Green I. Curcumin is a groove binding ligand (i.e., insertion of curcumin molecule in the voids within the double helix of the ssDNA), while SYBR Green I is an intercalator agent (i.e., intercalates between base pairs). Furthermore, curcumin weakly binds to the aptamer, while SYBR Green I strongly binds to the aptamer. These two parameters could explain the lower sensitivity obtained with curcumin. It was applied for the detection of Vitamin A and Bisphenol A as proofs of concept. It showed that the increase of sensitivity is not linked to only one aptamer and its specific sequence. Therefore, this new probe could also be exploited to develop novel aptasensors for the detection of antimicrobial residues.

**Figure 3:**
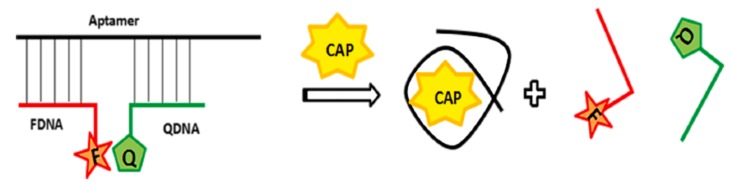
Schematic illustration of the structure-switching signaling aptamers used for chloramphenicol (CAP) detection [80]. Reprinted [80]; with permission from Elsevier.

The most common format of aptasensor is the use of a fluorescence-labeled aptamer that is a combination of one aptamer labeled with a fluorophore and a quencher (Figure 3). The fluorescence emitted by the fluorophore (e.g., fluorescein, FAM, cyanine) is quenched in the presence of nanomaterials, such as carbon nanotubes (CNTs), carbon nanoparticles (CNPs), graphene oxide (GO), MoS_2_ nanostructures, gold nanoparticles (AuNPs), quantum dots (QDs), or composite materials.

Fluorescence resonance energy transfer (FRET) assay is based on the energy transfer between two fluorescent molecules (a donor and an acceptor, such as fluorophore and quencher, respectively). The consequence is a strong fluorescence quenching. When the quencher is far from the fluorescent probe, the fluorescence is increased. On the contrary, when the quencher is close to the fluorescent probe, the fluorescence is quenched. The advantages of FRET-based aptasensors are their rapidity, high sensitivity, good selectivity, and little or no pollution [80].

**Figure 4:**
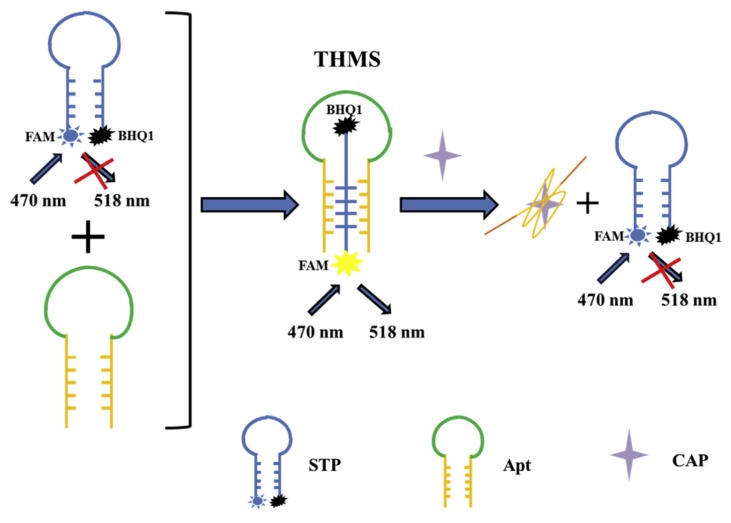
Schematic illustration of the aptamer-based triple-helix molecular switch for the detection of chloramphenicol [79]. Reprinted from [79]; with permission from Elsevier.

Two major types of fluorescent detection use metal nanoclusters for quenching purposes: “turn-off” mode (fluorescence quench induced by the presence of the target) and “turn-on” mode (fluorescence increases in the presence of the target). For instance, aptamers with a hairpin structure can be labeled with a fluorescent compound and a quenching dye at the 5′ and 3′ end of the aptamer (named aptabeacon), respectively (Figure 4). Quencher and fluorophore are in close vicinity in such configuration. Therefore, the fluorescence is quenched. The change of conformation of the aptamer in the presence of the target will tend to increase the distance between the fluorophore and the quencher, and, as a consequence, the fluorescence will tend to increase. This is a “turn-on” aptasensor.

In recent years, triple-helix molecular switch (THMS)-based aptasensors have been used for food analysis [111]. For instance, Tu et al. (2020) developed a THMS-based aptasensor for the detection of chloramphenicol in honey samples (Figure 3) [79]. This method is based on the formation of the triple-helix molecular switch (THMS) between a signal transduction probe (STP) and a label-free hairpin-shaped aptamer with two arms segments acting as a recognition probe. The STP is a dual-labeled oligonucleotide (labeled with a fluorophore and a quencher at the 3′ and 5′-end). When the STP and the aptamer form the THMS structure, the fluorescence can be emitted. Therefore, when the target analyte is absent, the THMS structure is formed, and the fluorescence increases. On the contrary, the structure cannot be formed when the target analyte is present. In this case, the fluorescence is quenched because the fluorophore and the quencher are in close vicinity in the dual-labeled oligonucleotide. This technique is interesting when compared to the molecular beacon and double-helix molecular switches because, in this case, the aptamer is not labeled, and, therefore, its affinity and specificity are preserved. Furthermore, the THMS structure is very stable.

The use of nanomaterials has led to great improvements in the performance of aptasensors in terms of sensitivity [112,113]. Typical nanomaterials, such as carbon dots (CDs) and gold nanoparticles (AuNPs), are often employed as quenchers in FRET-based aptasensors [77]. More recently, metal-organic frameworks (MOFs) have been exploited because of their highly efficient quenching properties [85]. Upconversion nanoparticles (UCNPs) are a class of optical nanomaterials doped with lanthanide ions as an activator. UCNPs can be used as highly sensitive label reagents. Wu et al. (2015) combined cDNA to UCNPs, emitting a fluorescent signal for the detection of CAP in milk [99]. When the target is absent, cDNA binds to the aptamer, and the fluorescence emitted by UNCPs is maximal. When the target is present, the complex UCNPs-cDNA is released from the aptamer-conjugated magnetic nanoparticles. Therefore, the fluorescence decreases. MNPs are often used for both recognition and concentration of the target analyte. The authors have reported a detection limit as low as 0.01 ng/mL.

**Figure 5:**
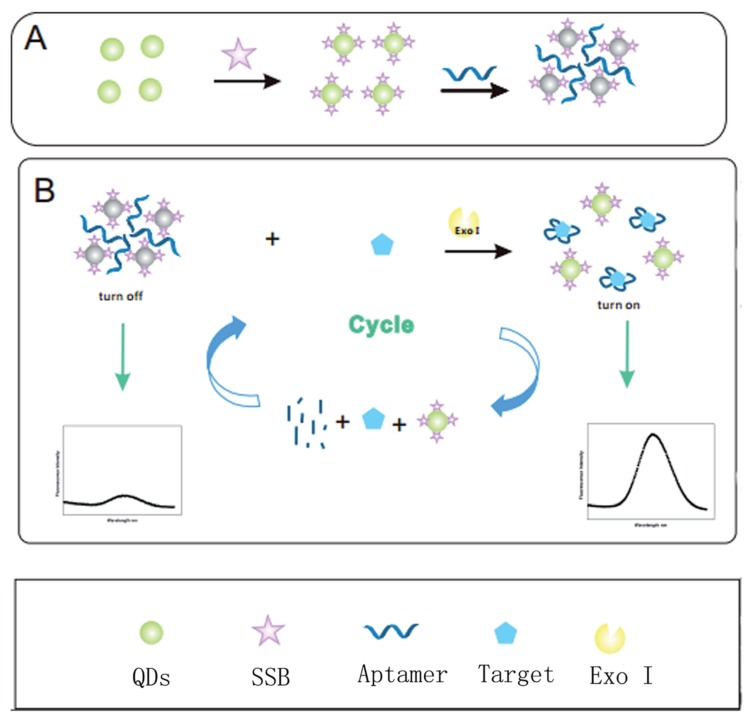
Schematic illustration of the label-free fluorescent probe based on structure-switching aptamers for streptomycin detection [95]. (**A**) Synthesis of fluorescent switch probe; (**B**) Schematic of fluorescent switch aptasensor detection of streptomycin based on single-stranded DNA binding protein labeled quantum dot with exonuclease-assisted target recyclings. Reprinted from [95]; with permission from The Royal Society of Chemistry.

Quantum dots (QDs) are innovative nanocrystalline semiconductors (10 to 100 Å) (e.g., cadmium telluride quantum dots (CdTe QDs), nitrogen-doped graphene quantum dots (N-GQDs) [114]). They are constituted of a semiconductor core coated by a semiconductor polymer shell [115]. Stanisavljevic et al. (2015) presented QDs as the new fluorophores because of their interesting optical properties (e.g., higher fluorescence, photostability, multicolor QDs) [116]. QDs have been used directly or in conjugation with a biosensing element (i.e., aptamer) for the detection of pesticides and veterinary drug residues [117]. QDs have been used several times as quenchers in FRET-based aptasensors for the detection of antimicrobial residues in food products [83,86,87]. Wu et al. (2017) developed an aptasensor for the detection of streptomycin in milk [95]. The signal can be amplified, and so the sensitivity increases, by using exonuclease-assisted target recycling technique (Figure 5). The aptamer binds first to the single-strand DNA binding protein (SSB) grafted on quantum dots (QDs). QDs tend to aggregate in solution, which leads to the self-quenching effect of QDs. However, in the presence of the target, the aptamer preferentially binds to the target, resulting in the release of QDs and the increase of fluorescence signal. Further use of Exonuclease Exo I to digest the complex aptamer-target will result in the release of the target in the solution for a new binding cycle with other aptamers.

**Figure 6:**
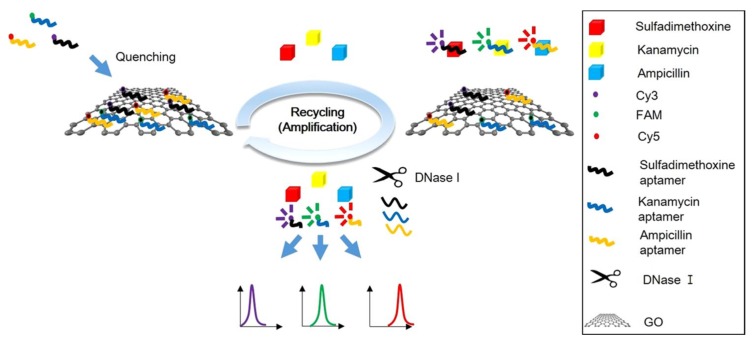
Schematic illustration of a high-efficient multiplex graphene oxide (GO)-based aptasensor for the detection of antibiotics using cyclic enzymatic signal amplification (CESA) with DNase I [47]. Reprinted from [47]; with permission from Springer Nature (http://creativecommons.org/licenses/by/4.0/).

The multiplex detection of authorized antimicrobials in food products is of great importance. Youn et al. (2019) developed a multiplex FRET-based aptasensor for the multiplex detection of three antimicrobials (sulfadimethoxine, kanamycin, and ampicillin) in milk (Figure 6) [47]. The multiplexing strategy is based on the combined use of three different aptamers, specific for sulfadimethoxine, kanamycin, and ampicillin, labeled with three different fluorophores (cyanine 3 (Cy3), 6-carboxyfluorescein (FAM), and cyanine 5 (Cy5)), quenched by graphene oxides (GO). Furthermore, to improve the sensitivity of the aptasensor, a DNase I-assisted cyclic enzymatic signal amplification (CESA) method is used. DNase I is a non-specific endonuclease that cleaves single-stranded DNA. Graphene oxides are protective elements towards DNase I. The fluorophore-labeled aptamers are non-covalently bound to GO. Therefore, when the antibiotic is added and binds to the aptamer, the fluorophore-labeled aptamers are released in solution and digested by DNase I. The digestion leads to the recycling of antibiotics that can again bind to another fluorophore-labeled aptamer. The recycling of antibiotics allows the amplification of the fluorescence signal and improves the sensitivity of the aptasensor.

Liu et al. (2015) developed a fluorescent aptasensor for the detection of oxytetracycline and kanamycin, based on two aptamers and two signal labels (i.e., two fluorophores: fluorescein amidite or carboxyfluorescein (FAM) and carboxy-X-rhodamine (ROX)) for multiplexing purposes [105]. Both aptamers are immobilized onto magnetic nanoparticles (MNPs). Two different cDNA are conjugated, each one with a different fluorophore, one with green FAM (520 nm), and one with yellow ROX (608 nm). When both targets (oxytetracycline and kanamycin) are missing, both cDNA can bind to the dual aptamers-MNPs. Therefore, the fluorescent signal reaches maximum intensity at the two wavelengths (520 and 608 nm). When one of the targets is present, it binds with its specific aptamer. Therefore, fluorophore-labeled cDNA is released. The fluorescence signal intensity emitted by this fluorophore declines. With such molecular configuration, it is possible to discriminate between both antimicrobials and to conclude which one is present in a sample. Limits of detection have been set as low as of 0.85 ng/mL and 0.92 ng/mL for kanamycin and oxytetracycline (OTC), respectively.

### 3.2. Colorimetric Aptasensors

**Figure 7:**
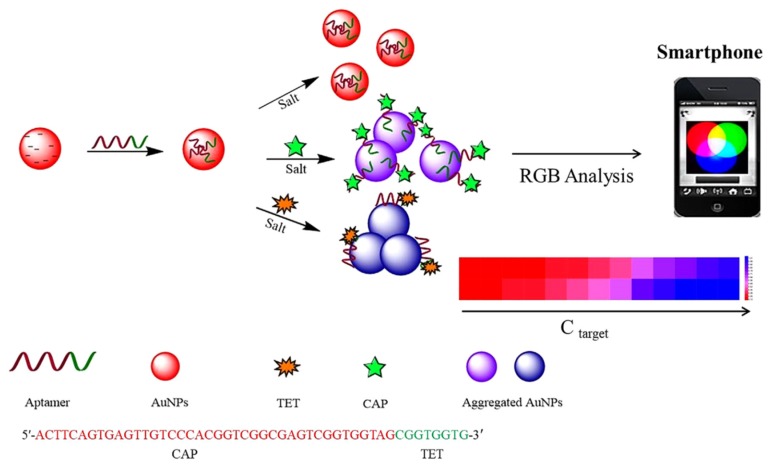
Schematic illustration of the multiplex detection of tetracycline (TET) and CAP based on gold nanoparticles (AuNPs) colorimetric aptasensors. Color changes can be detected by UV-spectrum and Smartphone analysis, respectively [50]. Reprinted [50]; with permission from Elsevier.

The second type of aptasensors that was developed during the 10 past years for the detection of antimicrobial residues are colorimetric aptasensors. Their advantages are their low cost and the possibility to read the results visually sometimes (simply using the naked eye) or with a smartphone (easy to export the data). Naked eye detection, as well as reading with smartphones, presents a great advantage to develop simple, quick, and cheap aptasensors for field-testing, on-site applications, and self-control in food industries. Wu et al. (2020) developed a colorimetric aptasensor that uses a smartphone for reading (Figure 7) [50]. The detection limits in the buffer are calculated as low as 7.0 nM and 32.9 for tetracycline (TET) and CAP, respectively.

In the past, colorimetric assays often lacked sensitivity. The use of advanced nanomaterials (e.g., gold nanoparticles (AuNPs), magnetic beads (MBs)) has improved their sensitivity [118].

Different types of colorimetric aptasensors have been designed based on Horseradish peroxidase (HRP) or HRP-mimicking DNAzyme and some others based on induced-aggregation of AuNPs [109]. Examples of colorimetric aptasensors developed for the detection of antimicrobial residues in animal-derived food products are presented in Table 2.

**Figure 8:**
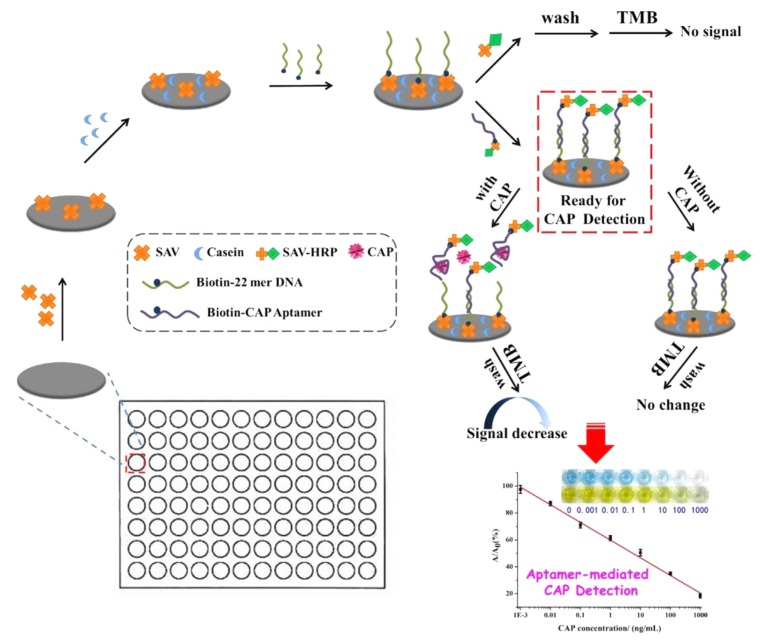
Schematic diagram for rapid aptamer-mediated colorimetric assay of CAP [44]. Reprinted from [44]; with permission from Elsevier.

• Colorimetric aptasensors based on natural HRP:

Yan et al. (2018) designed a simple colorimetric aptasensor for the detection of chloramphenicol (CAP) in honey and fish, based on HRP covalently bound to a specific aptamer pre-immobilized on a microtiter plate by hybridization with a complementary DNA (cDNA) (Figure 8) [44]. When CAP interacts with the specific aptamer, the aptamer-HRP conjugate is released from the plate and removed by washings. The enzyme HRP catalyzes the conversion of the chromogenic substrate (e.g., 3,3′,5,5′-tetramethylbenzidine (TMB)) into colored compounds in the presence of hydrogen peroxide (H_2_O_2_). The detection limit is calculated at 0.0031 ng/mL. Xu et al. (2018) developed a multiplex colorimetric aptasensor for the detection of oxytetracycline and kanamycin in milk combining HRP for colorimetric detection and AuNPs for signal amplification [119]. The aptamers are hybridized with cDNA (complementary probe) immobilized on magnetic beads (MBs). In the presence of one target, the aptamer is released, and so the cDNA is free to hybridize with the HRP-signal probe immobilized on AuNPs. In this case, HRP can catalyze the conversion of substrates. Two different substrates (TMB and o-phenylenediamine (OPD)) are used to simultaneously detect OTC and kanamycin and discriminate one from the other (when OTC is added, it changes from transparent to deep blue; when kanamycin is added, it changes from transparent to deep yellow).

**Figure 9:**
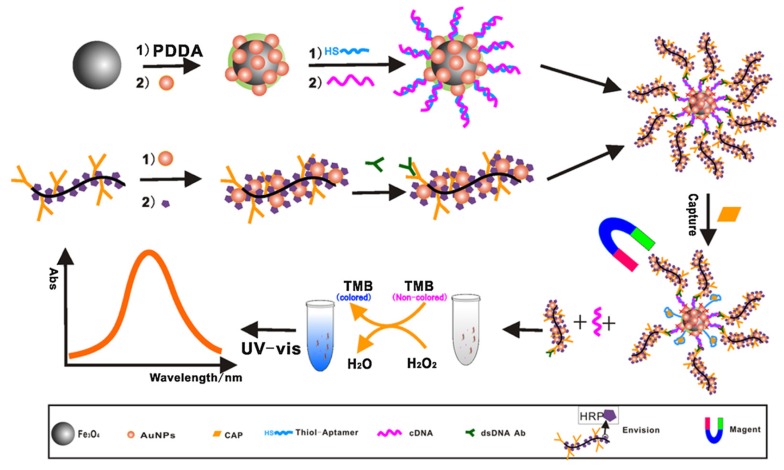
Schematic representation of a colorimetric aptasensor to detect CAP using ds-DNA Ab/EV-AuNPs-HRP as a signal tag [120]. Reprinted from [120]; with permission from Elsevier. EV: EnVision; HRP: horseradish peroxidase.

The polymer reagent (EnVision™ (EV)) that contains 100 HRP per polymer chain can be used to amplify the enzyme-linked chromogenic reaction to improve the sensitivity of aptasensors. Miao et al. (2015) developed an aptasensor, employing EV reagent immobilized onto magnetic nanoparticles (AuMNPs) (Figure 9) [120]. A double-strand (ds)-DNA antibody is co-immobilized with the enzymes. In the absence of the target, the CAP aptamer is hybridized with its complementary DNA (cDNA) to form the ds-DNA, which is recognized by the ds-DNA antibody. The EV reagent also contains a secondary antibody, which is able to recognize the ds-DNA antibody. The signal amplification is linked to both a high number of enzymes in EV reagent and a large surface of immobilization of MNPs. Similarly, signal amplification can also be obtained using the reagent PowerVision™ (PV) as a nano tracer because PV also contains many HRP enzymes [121].

**Figure 10:**
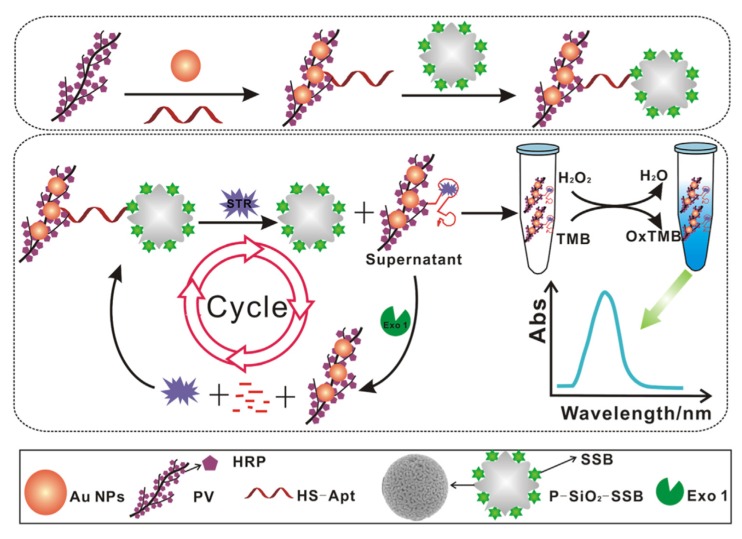
Schematic representation of biosensing of streptomycin dependent on the developed colorimetric aptasensor [121]. Reprinted from [121]; with permission from Elsevier.

**Figure 11:**
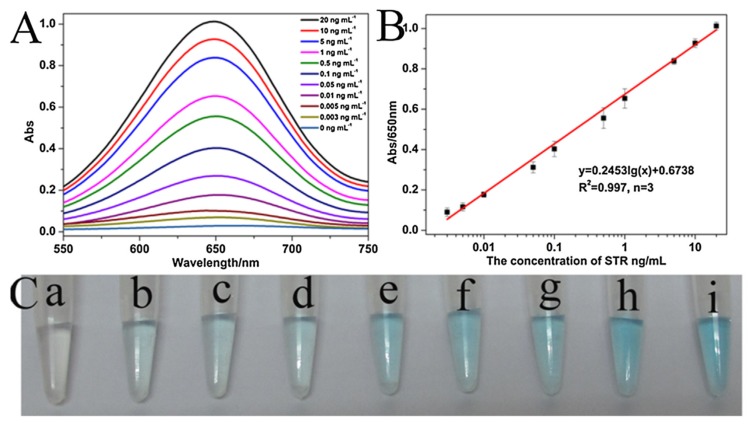
(**A**) UV–vis absorption spectra of the aptasensor in the presence of different concentrations of streptomycin (0, 0.003, 0.01, 0.1, 0.5, 1, 5, 10, 20 ng/mL), (**B**) The relationship between streptomycin concentration and UV absorbance intensity at 650 nm with the developed colorimetric sensor, (**C**) The corresponding digital camera pictures of colorimetric responses (from a to i: 0, 0.003, 0.01, 0.1, 0.5, 1, 5, 10, 20 ng/mL of streptomycin) [121]. Reprinted from [121]; with permission from Elsevier.

Luan et al. (2017) combined two ways of signal amplification: the use of PV reagent and exonuclease-assisted target recycling (Figure 10) [121]. The aptamer is bound to HRP (PV reagent) and metallic NPs (AuNPs) (Apt-Au-PV). When the target is present in solution, the aptamer-PV conjugate preferentially binds to the target, and AuNPs are released. Exonuclease I is added, which digests the aptamer, and so releases the target and PV reagent that are ready for a new cycle of reaction with aptamers and AuNPs. These repeated cycles lead to signal amplification. Due to the amplification systems (a combination of PV and exonuclease-assisted target recycling), the sensitivity is improved. Streptomycin can be quantified in milk samples using the linear relationship between streptomycin concentrations and UV absorbance intensity at 650 nm (Figure 11A,B). The detection limit is as low as 1 pg/mL with the instrumental reading. Furthermore, the color differences between different streptomycin concentrations can be distinguished with the naked eye (Figure 11C).

**Figure 12:**
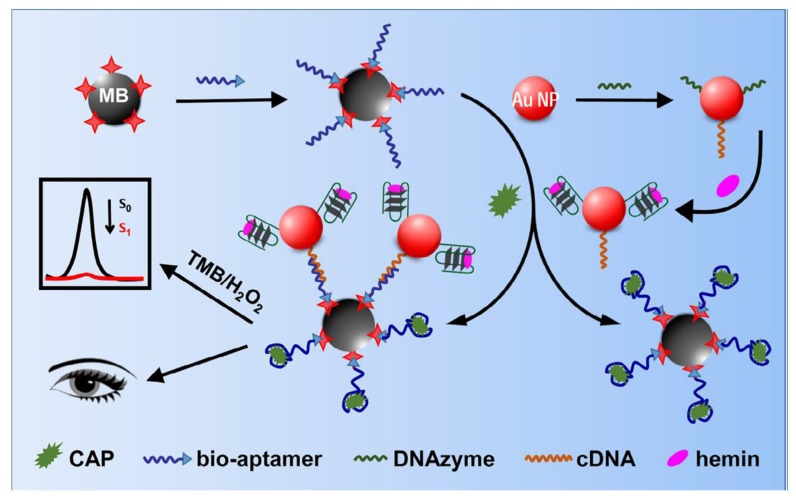
Schematic representation of the competitive aptasensing of CAP based on the colorimetric signal transduction with the hemin/G-quadruplex DNAzyme-functionalized AuNPs nanoprobe [131]. Reprinted from [131]; with permission from Elsevier.

• Colorimetric aptasensors based on HRP-mimicking DNAzymes: 

As an effective alternative to the natural enzyme, the use of HRP-mimicking DNAzymes (i.e., nucleic acids with catalytic properties) is now getting more interest. The peroxidase-like activity of DNAzymes can be detected with a colorimetric output signal. One big advantage of DNAzymes is that there is no need to produce HRP-conjugates (i.e., antibody-HRP or target-HRP conjugates) that can be easily altered. Therefore, the hemin/G-quadruplex DNAzymes are extensively used in optical and electrochemical biosensors to detect a broad range of targets [144]. The principle of detection of DNAzyme-based aptasensors relies on the activity of a DNAzyme that is blocked by a cDNA. When the target is present, the aptamer binds to the target and its conformation changes. Then, the cDNA is released from the DNAzyme, which recovers its enzymatic activity. The oxidization of the substrate by the DNAzyme produces the colorimetric signal. Cui et al. (2018) developed a colorimetric aptasensor based on HRP-mimicking DNAzyme (hemin/G-quadruplex) for the detection of kanamycin in milk [123]. The G-quadruplex structure bound with hemin (ferric chloride heme) becomes an active HRP-mimicking DNAzyme, in the presence of alkali metal cations (e.g., potassium). The formation of the G-rich structure is modulated by the target. Huang et al. (2019) developed a colorimetric aptasensor for the detection of CAP in milk powder based on the aptamer-conjugated magnetic beads for the recognition of the target and the combination of gold nanoparticles and hemin/G-quadruplex DNAzymes for the detection (Figure 12) [131]. Then, the colorimetric signal is amplified due to the high surface of AuNPs for the immobilization of many hemin/G quadruplex DNAzymes.

**Figure 13:**
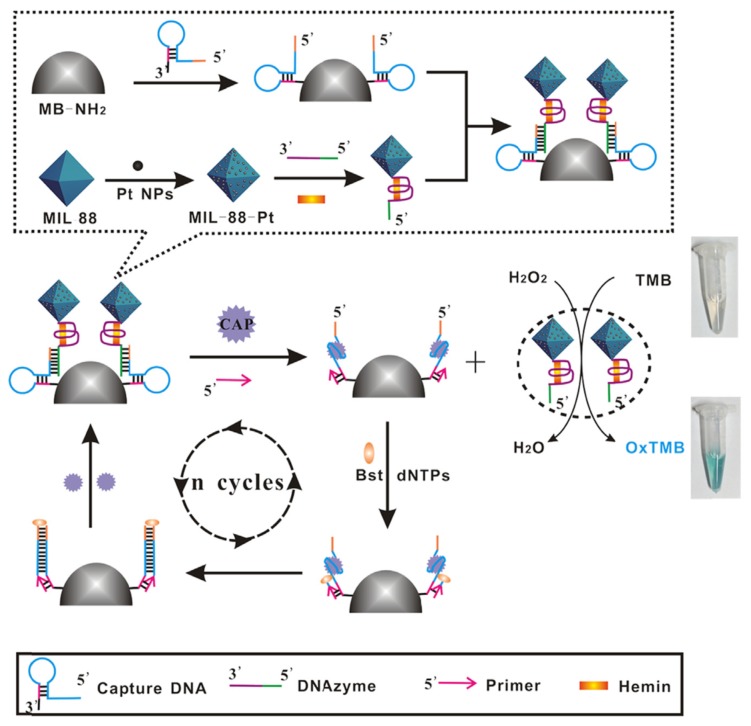
Schematic representation of the colorimetric sensor for CAP detection based on the single-stranded DNAzyme-labeled MIL-88-Pt and circular strand-displacement polymerization (CSDP) for signal amplification [130]. Reprinted from [130]; with permission from Elsevier.

Luan et al. (2018) developed a colorimetric aptasensor for the detection of CAP in milk, based on a novel tag, DNAzyme-labeled Fe-MIL-88-Pt (Figure 13) [130]. Firstly, hairpin cDNA containing the aptamer sequence is immobilized onto magnetic beads (MBs). Secondly, the signal tag is composed of three elements, single strand DNAzyme (G-quadruplex/hemin structure), platinum nanoparticles, and MIL-88 that all together contribute to the catalysis of the substrate tetramethylbenzidine (TMB) in the presence of H_2_O_2._ The catalytic activity is amplified because the three elements possess a peroxidase-like activity. In the absence of the target, the DNAzyme-labeled Pt-MIL88 can hybridize with its cDNA contained in the hairpin DNA-labeled MBs. In this case, the DNAzyme is not free to catalyze the oxidation of the substrate TMB. There is no change in color. Conversely, when the target (CAP) is present, the DNAzymes-labeled Pt-MIL-88 are released, followed by the TMB substrate oxidation and the generation of a blue color. Furthermore, target-triggered circular strand-displacement polymerization (CSDP) has been used for signal amplification [145]. CSDP is based on the lengthening of an oligonucleotide primer based on Bst DNA polymerase, to replace the target (CAP), and thus release it to initiate a new cycle.

**Figure 14:**
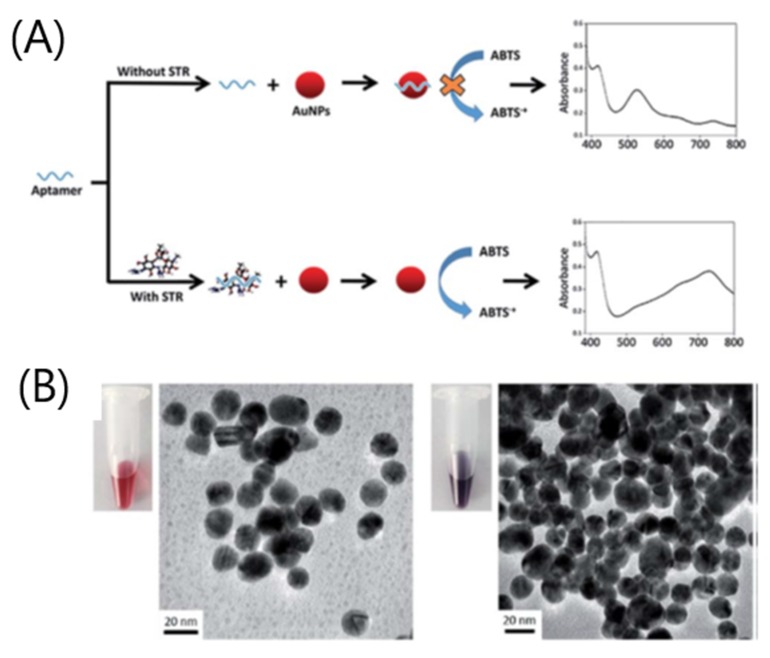
(**A**) Schematic illustration of the designed aptasensor for streptomycin (STR) detection utilizing enhanced catalytic activity of AuNPs. (**B**) TEM images and color of AuNPs solutions with and without streptomycin. Left: 50 nM STR1 aptamer + AuNPs; right: 50 nM STR1 aptamer + 1 mM STR + AuNPs [125]. Reprinted from [125]; with permission from The Royal Society of Chemistry.

• Colorimetric aptasensors based on peroxidase-like activity of Au nanoparticles (or nanozymes): 

More recently, AuNPs are the most widely used nanomaterials in colorimetric aptasensors. Different characteristics of NPs are exploited to develop AuNPs-based optical aptasensors, but with different modes of detection. The first exploited characteristic is the intrinsic peroxidase-like activity of AuNPs. Zhang et al. (2020) developed a very simple colorimetric aptasensor for the detection of tetracycline in milk [138] based on the intrinsic peroxidase-like activity of gold nanoclusters (AuNCs). The aptamer is combined with AuNCs for two main reasons. Firstly, the aptamer is specific for the detection of tetracycline. Secondly, the aptamer that could bind onto the surface of AuNCs tends to improve their catalytic activity. When tetracycline is absent, the enhanced peroxidase-like activity of aptamer-labeled AuNCs (Apt-AuNCs) oxidizes the substrate TMB to form an intense blue product. When tetracycline is present in solution, the aptamer that binds to the target will alter its conformation. As a consequence, the interaction between the aptamer and AuNCs is modified, the capacity of oxidation is reduced, which leads to a lighter blue color. The signal is inversely proportional to the target concentration. On the contrary, Zhao et al. (2017) developed a colorimetric aptasensor for the detection of streptomycin in milk, based on the inhibition of oxidative capacity (i.e., peroxidase-like activity) of gold nanoparticles when the aptamer is adsorbed onto their surface [125]. Therefore, in the absence of the target, the aptamer is adsorbed onto the surface of AuNPs, which inhibits the catalytic activity of AuNPs and causes limited substrate oxidation (Figure 14). When the target is present, the aptamer binds to the target instead of AuNPs, which, in turn, recover their oxidative capacity. The substrate 2,20-azino-bis(3-ethylbenzothiazoline-6-sulfonic acid) (ABTS) is usually oxidized to generate a green product. The absorbance value (at 733 nm) is proportional to the streptomycin concentration. Finally, it is noteworthy that the complex streptomycin-aptamer tends to enhance the oxidative capacity of AuNPs, which leads to signal amplification.

• Colorimetric aptasensors based on size-dependent colors of AuNPs:

In this case, the principle of colorimetric detection is based on AuNPs characteristics. The second interesting characteristic of AuNPs is size-dependent colors (dispersed AuNPs: red color, aggregated AuNPs: blue/purple color), high extinction coefficients (>3 orders of magnitude times larger than those of organic dyes [139]). This change of color is visible by the naked eye. Two different types of aptasensors based on AuNPs can be developed: (i) label-free and (ii) labeled aptasensors.

Label-free aptasensors can be based on target-specific and salt-induced aggregation of unmodified AuNPs. However, aptasensors based on the aggregation of NPs in saline solution are sensitive to environmental conditions (e.g., acidity, ionic strength). Therefore, they are more likely to give false-positive results. In label-free aptasensors, aptamers are physically adsorbed onto unmodified AuNPs, preventing the aggregation of AuNPs. In the presence of the target, the aptamers are released from the AuNPs that are no more protected, and so the AuNPs aggregation leads to a change of color (i.e., from red to purple/blue). Luo et al. (2015) developed a label-free colorimetric aptasensor for the detection of tetracycline in milk [139]. Cysteamine-stabilized gold nanoparticles (CS-AuNPs) are positively charged, while aptamers (polyanionic DNA) are negatively charged. The attraction between aptamers and NPs leads to the aggregation of NPs; there is no salt-induced aggregation in this case. However, the interaction between the aptamer and its target is stronger than the electrostatic interaction of the aptamer with CS-AuNPs. Therefore, when the target is present in solution, the aptamer binds to its target and prevents the aggregation of the released CS-AuNCs. The color change is ranging from purple/blue to red.

**Figure 15:**
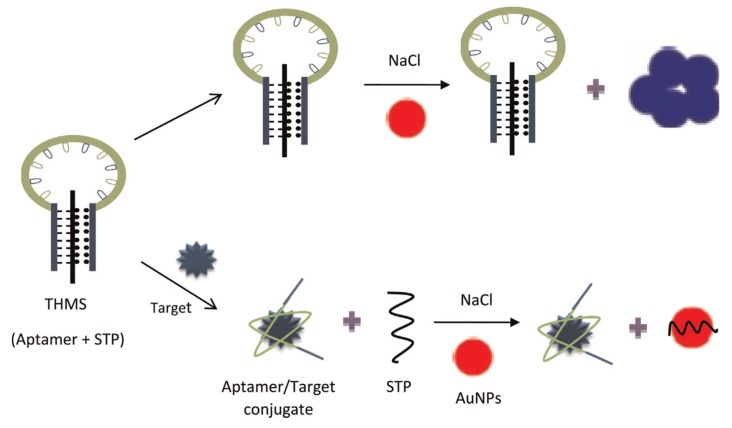
Schematic description of tetracycline detection based on the colorimetric triple-helix molecular switch (THMS) [140]. Reprinted from [140]; with permission from Elsevier.

As it was previously seen in fluorescence aptasensors, triple-helix molecular switch (THMS) is also used with colorimetric detection for signal amplification. Ramezani et al. (2015) developed a label-free colorimetric aptasensor that uses gold nanoparticles (AuNPs) for the colorimetric detection associated with THMS as a signal amplifier (Figure 15) [140]. The THMS structure is formed by the aptamer hybridized with the signal transduction probe (STP) (i.e., label-free STP). The THMS structure is stable when the target is absent. So, the salt-induced (i.e., NaCl) AuNPs aggregation can occur (blue color). In the presence of the target, the STP is released because the aptamer binds to its target, followed by its adsorption onto AuNPs, leading to their dispersion (red color).

Wu et al. (2020) developed a label-free colorimetric aptasensor for the multiplex detection of CAP and oxytetracycline (OTC) in chicken meat and milk, based on salt-induced gold nanoparticles (AuNPs) aggregation (Figure 7) [50]. In this setting, the capture probe is a multifunctional aptamer formed of two specific aptamers, one for each target, adsorbed onto AuNPs. When the target is absent, the aptamers will tend to adsorb onto the surface of AuNPs, and the aggregation of nanoparticles will be prevented. When one of the targets is present, it binds to its specific aptamer and causes the release from the AuNPs. It is noteworthy that the salt-induced aggregation of AuNPs can occur. Because the two aptamers for CAP and OTC are formed of fragments of different lengths (i.e., the length fragment for OTC is a fifth of that for CAP), their binding affinities to nanoparticles can be different (i.e., shorter sequence (OTC), stronger affinity). Consequently, different color changes can be observed, depending on the nature of the target that is present in the solution. Label-free aptasensors are easy to prepare, cost-effective, and time-saving.

Labeled aptasensors based on AuNPs modified with aptamers or their specific probes are developed [126]. The aptamer can be first covalently bound onto the AuNPs. Then, a further step is to purify modified AuNPs by separating them from unmodified AuNPs and free aptamers. This extra step is time-consuming, increases the cost, and can modify the affinity of the aptamer towards its target. In the presence of the target, the target binds to the AuNPs-labeled aptamer, and so the aggregated AuNPs are dispersed, changing the color from purple to red. Lateral flow devices (LFD) (i.e., dipsticks) are simple immunoassay systems intended to detect the presence of a target analyte in a sample. This principle can be applied to the fabrication of dipsticks, by the immobilization of aptamer-linked NPs aggregates onto the pad (i.e., cellulose membrane) [31,146]. Aptamer-linked NP aggregates are spotted on the conjugation pad, while streptavidin is spotted as a thin line. When the target analyte is present, it binds to the aptamer, and so the NP aggregates disassemble. Dispersed biotinylated nanoparticles can migrate along the membrane and be captured by streptavidin. Then, a red line appears. LFD is much more convenient and more sensitive aptasensors than colorimetric assays in solution, especially for point of care (POC) testing. It can be applicable to the detection of antimicrobial residues in foodstuffs. When an aptamer binds covalently to AuNPs, a conformational change of the aptamer occurs in the presence of the target, it then induces the aggregation of AuNPs in a salt solution, and subsequently causes a change of color from red to purple [126]. Abnous et al. (2018) used a further approach when they have developed an aptasensor based on aptamers that bind to AuNPs and specific DNA probes [147]. In such settings, aptamers would hybridize with their complementary ssDNAs, and the newly formed dsDNA would stabilize the AuNPs that cannot aggregate anymore, and hence the color would stay red. In the presence of the target, the DNA probe and the aptamer would be released because the aptamer tends to bind preferentially to its specific target. Consequently, the AuNPs would aggregate, and the color would turn to blue.

Instead of using NPs, aptamers can bind to the surface of a conducting colored polymer (polydiacetylene (PDA)) liposomes. The PDA liposomes change their color by external stimuli, including ligand interaction, temperature, solvent, and pH. When the aptamer binds to the target, the color of liposomes changes [31].

**Figure 16:**
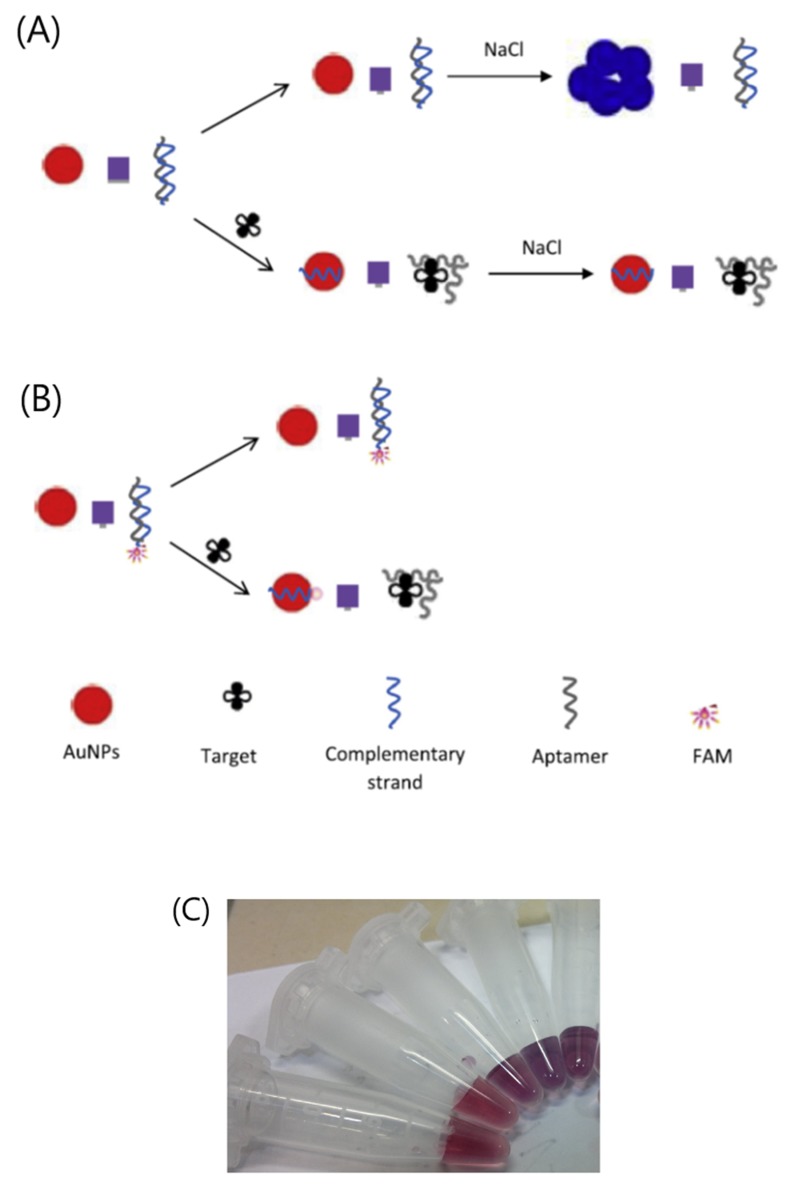
Schematic description of streptomycin detection based on (**A**) colorimetric and (**B**) fluorescence quenching aptasensors, (**C**) Visual color change upon treatment of AuNPs and dsDNA with different concentrations of streptomycin (0, 30, 300, 2000, 4000 nM, from right to left) [142]. Reprinted from [142]; with permission from Elsevier.

Song et al. developed a dual-detection method for the detection of ampicillin in milk [143]. Two modes of detection—colorimetric and fluorimetric—are simultaneously employed because AuNPs are both used as a quencher for the fluorescent reagent (FAM) and as a color signal probe due to the salt-induced aggregation/dispersion phenomenon of AuNPs. When ampicillin is absent in solution, the FAM-aptamer is adsorbed onto the AuNPs, and the fluorescence is quenched by AuNPs. The AuNPs are then dispersed, producing a red color signal. In the presence of ampicillin and salt (NaCl), the aptamer binds to its target and is desorbed from AuNPs. As a consequence, the fluorescence signal is recovered, and simultaneously AuNPs can aggregate to produce a color transition from red to purple/blue. The detection limits of fluorescence and colorimetric aptasensors range from 2 ng/mL to 10 ng/mL, respectively, for ampicillin in milk. Emrani et al. (2016) also proposed a dual-detection aptasensor for streptomycin in milk (Figure 16) [142]. The limits of detection for FRET and colorimetric aptasensors are equal to 47.6 and 73.1 nM, respectively. These two studies are in favor of using fluorescence aptasensors than colorimetric aptasensors because of lower detection limits.

### 3.3. Other Optical Aptasensors

Applications of other optical aptasensors for the detection of antimicrobial residues in animal-derived food products are presented in Table 3.

#### 3.3.1. Chemiluminescent (CL) Aptasensors

**Figure 17:**
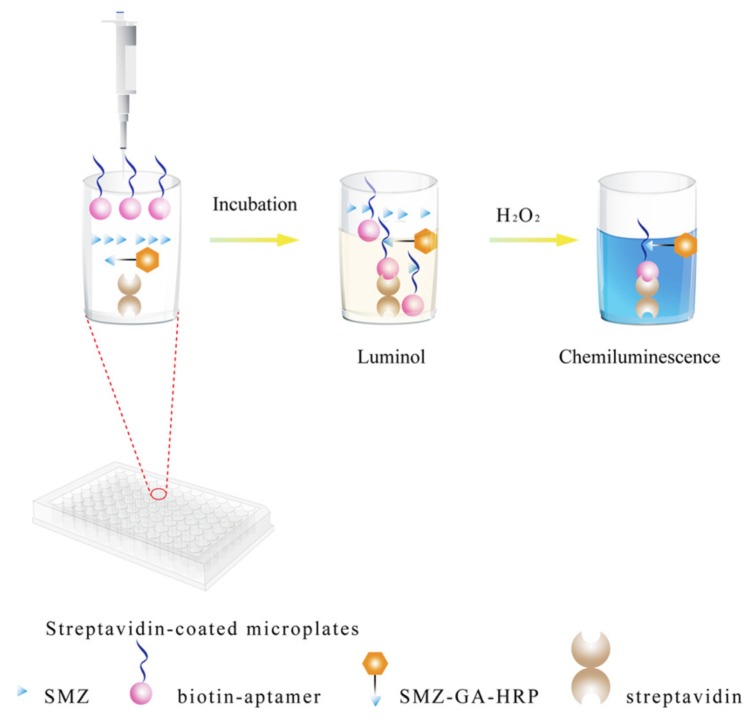
Schematic illustration of the chemiluminescent aptasensor for the detection of sulfamethazine (SMZ) in milk [148]. Reprinted from [148]; with permission from Elsevier.

A chemiluminescent aptasensor is based on a chemical reaction that produces light, usually in the visible or near-infrared regions. Luminol is one of the most widely used chemiluminescent reagents, N-(4-aminobutyl)-N-ethylisoluminol (ABEI) is another [49]. The oxidation of luminol reagent is catalyzed by the enzyme horseradish peroxidase (HRP) in the presence of hydrogen peroxide. This reaction produces the chemiluminescent signal. Yang et al. (2019) developed a chemiluminescent aptasensor for the detection of sulfamethazine (SMZ) in milk (Figure 17) [148]. It is based on the competition between SMZ, when present in solution, and a conjugate SMZ-HRP. When SMZ is present in solution, fewer conjugates are retained onto the surface of the microplate by the aptamer, and, therefore, the chemiluminescent signal decreases. The detection limit is determined to be as low as 0.92 ng/mL. Potassium ferricyanide or potassium periodate is often used as a catalyst for the oxidation of luminol.

As it is the case with fluorescence and colorimetric aptasensors, the use of nanomaterials in CL aptasensors allows for improvement in their sensitivity and selectivity. Nanomaterials can be used to accelerate the decomposition of luminol in the presence of hydrogen peroxide (e.g., AgNPs [151]) to increase the signal and, hence, to improve the sensitivity of the aptasensor. In addition to catalyzing the reaction (i.e., facilitation of the electron transfer), nanoparticles are good carriers for immobilizing aptamers. Electrochemiluminescence (ECL) combines the strengths of electrochemical and chemiluminescent detection.

Wang et al. (2019) summarized the optical and mechanical properties of carbon dots (CDs) [159]. CDs can act as emitting species, energy acceptor, or catalysts of the CL reaction, or even oxidants. The properties of CDs allow improving the sensitivity of chemiluminescent aptasensors.

Bonilla et al. (2016) discussed about the opportunities offered by quantum dots (QDs) (e.g., high photostability, higher luminescence than conventional dyes, water solubilization, easy differentiation of different QDs (different size and/or composition, covalent conjugation of antibodies, or aptamers to QDs)), their limitations (e.g., cytotoxicity, non-specific binding). They have presented some applications in food science and biology for the detection of pathogenic bacteria and proteins. QDs are innovative nanocrystalline semiconductors that can be used as chemiluminescent reagents. Under the same excitation light, depending on the QD composition (e.g., cation composition) and size, QDs can emit fluorescence in the visible range, at different emission wavelengths (e.g., 525 and 585 nm). This property is used to develop multiplex assays based on the measurement of luminescence intensity at both wavelengths [160]. AuNPs are used as quenchers of the emission of QDs. The emission of luminescence from QDs is increased in the presence of the target analyte. QDs can act as catalysts and can amplify luminescence response [161,162].

Luminescent metal complexes can be used instead of classical CL reagents (e.g., square-planar luminescent platinum(II) complex (SPLPt(II))) [150]. They present several advantages: long phosphorescence lifetimes, and large Stoke shift values that reduce self-quenching.

#### 3.3.2. Surface Plasmon Resonance (SPR) Aptasensors

The combination of aptamer recognition of antimicrobial residues and detection by surface plasmon resonance (SPR) is still very poorly reported in the literature. An aptasensor based on SPR detection was developed for the screening of neomycin B in the buffer, but no application in real food samples has been reported [152].

#### 3.3.3. Surface-Enhanced Raman Scattering (SERS) Aptasensors

SERS technology is based on the enhanced Raman scattering of the molecules adsorbed on, or near, the SERS-active surfaces (e.g., metallic nanostructures (gold or silver NPs)). SERS achieves significantly high sensitivity, rapidness, simple sample preparation, and ease of use. Tang et al. (2018) reviewed SERS sensors for the detection of small analytes (e.g., heavy metals, pesticides, antibiotics) and pathogens for food safety and environmental monitoring [163].

**Figure 18:**
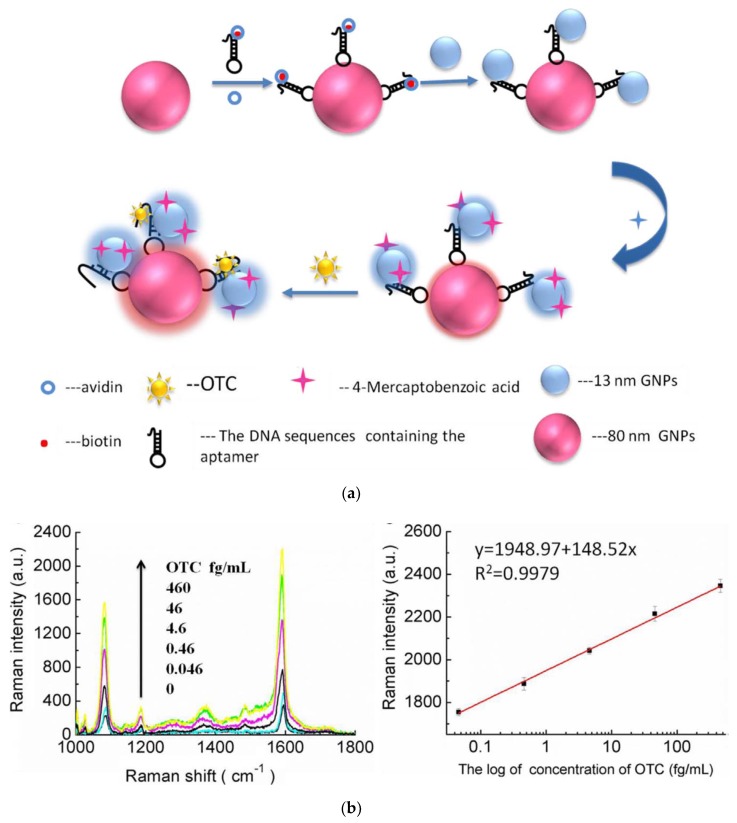
(**a**) Schematic diagram of nano-biosensor with surface-enhanced Raman scattering (SERS)-active for the detection of oxytetracycline (OTC); (**b**) the SERS spectra of different concentration of OTC and calibration curve of the SERS spectra of the 4-MBA at 1592 cm^−1^ for OTC detection [155]. Reprinted from [155]; with permission from Elsevier.

Meng et al. (2017) developed a SERS aptasensor for the detection of oxytetracycline in fishmeal [155]. This aptasensor is based on two kinds of gold nanoparticles with two different sizes (13 nm and 80 nm), linked together by a stem-loop DNA, comprising the aptamer and its complementary sequence (Figure 18). The Raman reporter molecule is 4-mercaptobenzoic acid (4-MBA). When the target is absent, the aptamer binds to its complementary sequence, forming the stem-loop DNA. Then, the 13 nm AuNPs are far from the 80 nm AuNPs; the SERS signal is at its minimal level. When the target is present, the aptamer binds preferentially to the target, which leads to a partial dehybridization. Therefore, the two sizes of AuNPs get closer, and the SERS signal (Raman intensity) is increased. A very low detection limit is reported for oxytetracycline in fishmeal at 4.35 × 10^−3^ fg/mL.

#### 3.3.4. Resonance Rayleigh Scattering Spectra (RRSS) Aptasensors

**Figure 19:**
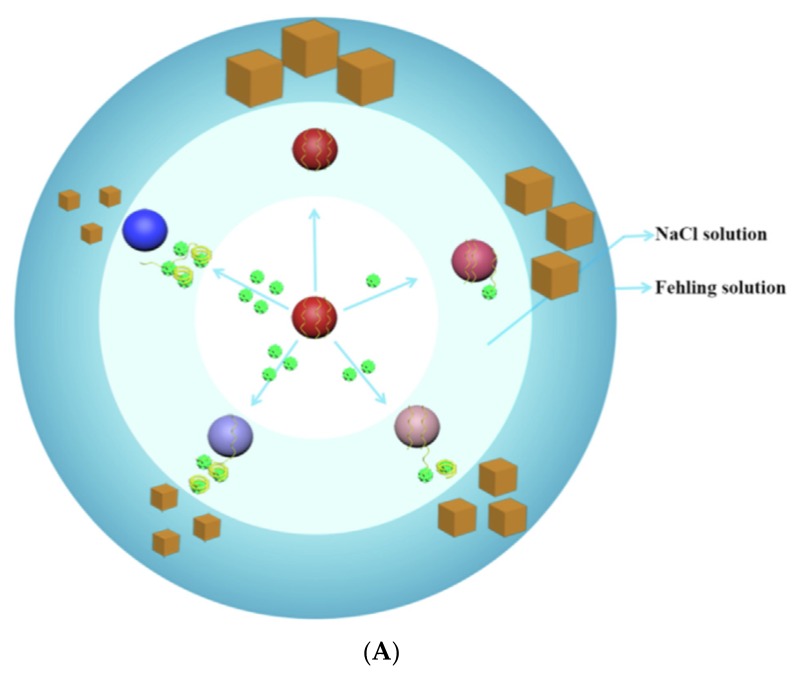

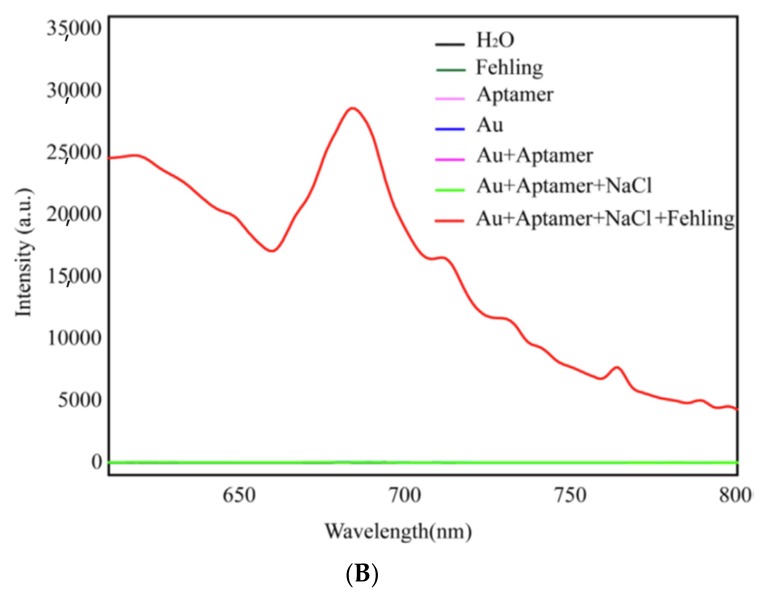
(**A**) Principle of the aptasensor for tobramycin based on Resonance Rayleigh scattering Spectra (RRSS); (**B**) RRSS of Fehling reagent catalytic system [158]. Reprinted from [158]; with permission from Elsevier.

Resonance Rayleigh scattering spectra (RRSS) is an inelastic form of light scattering. When the resonance scattering peak is located at or close to the molecular absorption band of the detected particle, Resonance Rayleigh scattering (RRS) is produced [164,165]. The RRS intensity is related to molecular size, shape, and quantity of analytes in solution [166]. When molecules aggregate in solution, the RRS signal is enhanced. Therefore, RRSS is a method used to investigate aggregate systems, which can be used for the detection of small molecules like antimicrobials [167]. Yan et al. (2019) developed an optical aptasensor for the detection of tobramycin in milk, based on aptamer for the binding to the target analyte and RRSS for the detection [158]. When the target is absent, the complexation of AuNPs with aptamers prevents the aggregation of NPs. By adding CuSO_4_ to the solution of AuNPs-aptamer, Yan et al. (2019) used the catalytic properties of dispersed AuNPs-Apt to reduce CuSO_4_ into Cu_2_O particles of different sizes (Figure 19). The sizes of Cu_2_O cubes depend on the concentration of AuNPs-Apt complexes in solution. When the target is absent, the sizes of Cu_2_O cubes are high, and the RRS signal is strong. When the target is present in solution, the aptamer binds to its target and is desorbed from AuNPs. Therefore, AuNPs can aggregate, and less AuNPs-Apt complexes are present in solution. The sizes of Cu_2_O cubes are smaller, and the RRS signal is lower. The combination of AuNPs with the detection mode RRSS allows improving the sensitivity of the developed aptasensor. The detection limit for tobramycin in milk is equal to 0.19 nM.

## 4. Conclusions and Perspectives

During the past decade, many research efforts have been directed towards the development of aptasensors for the detection of antimicrobial residues in food products. Most of such aptasensors exploit electrochemical and optical sensors. Among the optical aptasensors developed so far, fluorescence and colorimetric sensors have drawn a substantial interest. These aptasensors offer rapid response, high sensitivity, reliability, usually simple fabrication, and applicability to complex matrices like food products. The logical continuation of such sensors would be the development of POC testing systems for field applications as these could provide cost-effective, rapid, and sensitive self-control options.

Most of the aptasensors published were developed for the detection of chloramphenicol, aminoglycosides (e.g., kanamycin), and tetracyclines (tetracycline, oxytetracycline) as proofs of concept, probably because aptamers are currently available for these molecules. Nowadays, it is indeed quite challenging to find commercially available aptamers for a broader range of antimicrobial residues, despite their advantages. There is a combination of issues, impeding the widespread application of aptamers in the control of antimicrobial residues in foodstuffs. The reasons could be both technical (i.e., difficulty of aptamer production for small molecules, sometimes insufficient target-aptamer affinity, lack of standardized protocols) [52,53] and economical (i.e., small veterinary market, diagnostics market dominated by antibody-based assays (immunoassays) for decades, industries reluctant to invest capital in new developments to replace antibodies) [54]. Many published applications are for single analyte detection. These papers represent interesting initial proofs of concept that often exploit simple optical aptasensors with relatively high sensitivity but for one specific analyte only. However, multiplex detection of antimicrobial residues is most often an important requirement for field applications. Therefore, more aptamers are needed for the detection of antimicrobials, and especially, if manageable, generic aptamers are able to recognize a whole family of antimicrobials, instead of only one substance. When no generic aptamers are available, an alternative option would be to use several aptamers in parallel with different targets and different signal tags [47] or multifunctional aptamers [50]. However, the number of aptamers gathered in a single method would be probably limited. Furthermore, one aptamer could disrupt the interaction of another aptamer with its target. Finally, the cost of the assay would be increased in relation to the number of aptamers involved. Therefore, many efforts have to be directed towards the production of aptamers targeted to antimicrobial residues, reaching a compromise between high affinity and wide selectivity. The development of nanomaterial-based aptasensors for antimicrobial residues in animal-derived food should continue to incorporate multiplex screening methods.

This review shows that the sensitivity of aptasensors could be increased by different techniques: engineering of new aptamers with high affinities, using nanomaterials as signal probes (e.g., salt-induced aggregation of AuNPs) and/or for signal amplification (e.g., AgNPs used as a catalyzer to accelerate H_2_O_2_ decomposition). DNA amplification techniques are also implemented for signal amplification purposes (e.g., exonuclease-assisted target recycling, target-triggered circular strand-displacement polymerization (CSDP)). Furthermore, engineered structure-switching aptamer sensors (e.g., triple-helix molecular switch (THMS)) can be developed by coupling aptamers to a signal transduction probe (e.g., fluorophore) to generate a readout signal when the target binds to its specific aptamer. The development of nanomaterial-based biosensors with low detection limits for antimicrobial residues in animal-derived food should continue.

Many applications have been developed in milk because it is presumed to be the easiest food matrix to work with. However, even if it is a liquid matrix for which no extraction procedure is necessary, matrix effects could be high with milk (i.e., interferences with the analyte detection) and could disrupt the detection of antimicrobials. Furthermore, when extraction and/or clean-up procedures are needed, it would inevitably increase the time of analysis. It is of utmost importance to emphasize that most of the developed aptasensors proposed in the literature lack real and full validation, especially in complex matrices like food products. These aptasensors are usually developed and optimized for the detection of antimicrobials in a buffer. Furthermore, to support their methods, the authors usually have tested one or few real samples (e.g., milk, muscle, honey). The detection limits are most often determined in buffer conditions. These low detection limits (in the range of pg/mL) will not hold true when applied to real samples due to the introduction of inferences and variability in the matrix. These methods need to be very sensitive to achieve very low concentrations, sometimes below 1 ng/mL (or ng/g). Therefore, a lot of work is still needed to validate these methods in real conditions and according to current regulations (e.g., European regulation EC/657/2002 [5]).

## Figures and Tables

**Table 1:** Fluorescent aptasensors for the detection of antimicrobial residues in food products.

Detection Technique	Antimicrobial Family	Analyte	Food Product	Fluorophore/Quencher or Intercalating Dye * or Signal Labels	Limit of detection (LOD) (nM or ng/mL)	References
FRET	AMGL	Kanamycin	Milk	CDs/AuNPs	18 nM (10.5 ng/mL)	[72]
FRET		Kanamycin	Milk	CDs/l-MoS_2_ NSs	1.1 μM (640.9 ng/mL)	[73]
FRET		Kanamycin	Milk	FAM/rGO	1 pM (0.583 pg/mL)	[42]
FRET		Kanamycin	Milk	FAM/AuNPs (+ exonuclease III)	321 pM (0.19 ng/mL)	[74]
FRET		Kanamycin	Milk	BHQ1/GO QDs	6 pg/mL	[75]
FRET		Kanamycin	Milk	FAM/AuNPs	0.3 nM (0.175 ng/mL)	[76]
FRET		Neomycin B	Milk	FAM/AuNPs	0.01 μM (6.15 ng/mL)	[77]
FRET	BL	Benzylpenicillin	Milk	FAM/rGO	9.2 nM (3.1 ng/mL)	[78]
FRET	PHENI	CAP	Honey	FAM/BHQ1	1.2 nM (0.39 ng/mL)	[79]
FRET		CAP	Milk	FAM/BHQ1	0.70 ng/mL	[80]
FRET		CAP	Honey	FAM/BHQ1	0.285 pg/mL	[81]
FRET		CAP	Milk	DSAC_2_N/GO	1.26 pg/mL	[82]
FRET		CAP	Milk	CdTe QDs/GO	0.2 ng/mL	[83]
FRET		CAP	Milk, shrimp	FAM/ZrP MOF (PCN-222)	0.08 pg/mL	[84]
FRET		CAP	Milk, fish	SYBR Green I/Cu-TCPP MOFs NSs	0.3 pg/mL	[85]
FRET		CAP	Milk	QDs/AuNPs	3 pg/mL	[86]
FRET		CAP	Milk	QDs/AuNPs	0.3 pM (0.097 pg/mL)	[87]
FRET		Florfenicol	Milk	ATTO 647N/GO	5.75 nM (2.06 ng/mL)	[58]
FRET	QUINO	Ofloxacin	Milk	RB/AuNPs	4.61 nM (1.66 ng/mL)	[88]
FRET		Enrofloxacin	Milk	Enrofloxacin/GO	3.7 nM (1.33 ng/mL)	[89]
FRET	TETRA	OTC	Milk	FAM/BHQ1	6.44 nM (2.96 ng/mL)	[90]
FRET	SULFA	SDMX	Milk	FAM/CPNBs	10 ng/mL	[91]
FRET	MULTI	SDMX, kanamycin, ampicillin	Milk	Apt1-Cy3, Apt2-FAM, Apt3-Cy5/GO (+ DNase I)	1.997, 2.664, 2.337 ng/mL	[47]
Fluorescence	AMGL	Kanamycin	Milk	TO *	59 nM (34.4 ng/mL)	[92]
Fluorescence		Kanamycin	Milk	Thioflavin T + Apt + cDNA1 + cDNA2	0.33 nM (0.19 ng/mL)	[93]
Fluorescence		Streptomycin	Milk	SYBR Gold dye * (+ exonuclease III)	54.5 nM (31.7 ng/mL)	[94]
Fluorescence		Streptomycin	Milk	QDs (self-quenching)	0.03 ng/mL	[95]
Fluorescence	PHENI	CAP	Milk, fish	SYBR green *	0.033 pg/mL	[96]
Fluorescence		CAP	Fish	SSB/DIL-Lip vesicle	1 pM (0.32 pg/mL)	[97]
Fluorescence		CAP	Milk	BSA-AuNCs/CAP	33 nM (10.7 ng/mL)	[98]
Fluorescence		CAP	Milk	Apt- Fe_3_O_4_ MNPs + cDNA-UCNPs	0.01 ng/mL	[99]
Fluorescence	TETRA	OTC	Milk	Apt- Fe_3_O_4_ MNPs + cDNA-UCNPs	0.036 ng/mL	[100]
Fluorescence		Tetracycline	Milk	TO *	29 ng/mL	[101]
Fluorescence		Tetracycline	Milk, pork meat	UCNPs (+ Fe_3_O_4_ MNPs)	0.0062 ng/mL	[102]
Fluorescence		TTC, CTC, OTC, doxycycline	Milk	NMM + Apt + cDNA1 + cDNA2	4.6 ng/mL	[103]
Fluorescence	SULFA	Sulfadimethoxine	Fish	MNPs-Apt + NaYF4: Yb, Tm UCNPs-cDNA	0.11 ng/mL	[104]
Fluorescence	MULTI	CAP, kanamycin	Milk, fish	SYBR gold *	0.52 pg/mL, 0.41 pg/mL	[46]
Fluorescence		OTC, kanamycin	Pig muscle, milk, honey	Apt-MNPs + cDNA1-FAM + cDNA2-ROX	0.85 ng/mL, 0.92 ng/mL	[105]
FPIA	PHENI	CAP	Honey	FAM/GO/Streptavidin	0.5 pM (0.162 pg/mL)	[106]
FALIA	AMGL	Kanamycin	Milk	CNPs-Apt	5.10^−8^ ng/mL	[107]
SPEET + fluorescence	AMGL	Kanamycin	Milk	AuNPs-Apt + AgNCs	1 nM (0.58 ng/mL)	[108]

AgNCs, silver nanoclusters; AgNPs, silver nanoparticles; AMGL, aminoglycosides; Apt-MNPs, aptamer-modified magnetic nanoparticles; AuNPs, gold nanoparticles; BHQ1, black hole quencher 1; BL, beta-lactams; BSA-AuNCs, bovine serum albumin-stabilized Au nanoclusters; CAP, chloramphenicol; CDs, carbon dots; CdTe QDs, cadmium telluride quantum dots; CPNBs, coordination polymer nanobelts; CTC, chlortetracycline; Cu-TCPP, copper-tetrakis (4-carboxyphenyl) porphyrin; Cy, cyanine; DSAC_2_N, 9,10-bis{4-[2-(N,N,N-trimethylammonium)-ethoxy]-styrene}anthracene dibromide; FALIA, fluorescence-based aptamer-linked immunosorbent assay; FAM, fluorescein amidite or carboxyfluorescein; Fe_3_O_4_, Magnetite; FPIA, fluorescence polarization immunoassay; FRET, fluorescence resonance energy transfer; GO, graphene oxide; l-MoS_2_ NSs, layered MoS_2_ nanosheets; MNPs, magnetic nanoparticles; MOFs, metal-organic frameworks; MULTI, multiplex; NMM, N-methylmesoporphyrin IX; OTC, oxytetracycline; PHENI, phenicolated; QUINO, quinolones; RB, rhodamine B; rGO, reduced graphene oxide; ROX, carboxy-X-rhodamine; SDMX, sulfadimethoxine; SSB, single-stranded DNA binding protein; SDMX, sulfadimethoxine; SULFA, sulfonamides; SSB/DIL-Lip, SSB and DIL(1,1′-dioctadecyl-3,3,3′,3′-tetramethyl-indocarbocyanineper-chlorate) coimmobilized liposomes; TETRA, tetracyclines; TO, thiazole orange; TTC: tetracycline; UCNPs, upconversion nanoparticles; ZrP, zirconium-porphyrin.

**Table 2:** Colorimetric aptasensors for the detection of antimicrobial residues in food products.

Labeled or Label Free Detection	Antimicrobial Family	Analyte	Food Product	Detection Principle	Limit of detection (LOD) (ng/mL or nM)	References
Labeled	AMGL	Kanamycin	Milk	Apt-MBs + NMOF-Pt-sDNA	0.2 pg/mL	[122]
Label-free		Kanamycin	Milk	Hemin/G-quadruplex DNAzyme	14.7 pM (8.6 pg/mL)	[123]
Label-free		Kanamycin	Milk, meat	Intrinsic peroxidase-like activity of AuNPs	0.1 nM (58.2 pg/mL)	[124]
Labeled		Streptomycin	Milk	Apt-Au-PV	1 pg/mL	[121]
Label-free		Streptomycin	Milk	Intrinsic peroxidase-like activity of AuNPs	86 nM (50 ng/mL)	[125]
Labeled		Streptomycin	Milk, honey	AuNPs aggregation + Apt	100, 125 nM (58.2, 72.7 ng/mL)	[126]
Label-free		Tobramycin	Milk and chicken eggs	AuNPs aggregation + Apt + NaCl	23.3 nM (10.9 ng/mL)	[127]
Label-free	DYES	Malachite green	Fish	AuNPs aggregation + Apt + NaCl	15.95 nM (5.82 ng/mL)	[128]
Labeled	MULTI	OTC, kanamycin	Milk	HRP-AuNPs	1 ag/mL	[119]
Label-free		CAP, tetracycline	Chicken meat, milk	AuNPs aggregation + Apt	32.9 nM, 7.0 nM (10.6 ng/mL, 3.11 ng/mL)	[50]
Labeled	PHENI	CAP	Honey, fish	Aptamer-HRP	0.0031ng/mL	[44]
Labeled		CAP	Milk	Au MNPs-SSB + Apt-SiO2@Au-HRP	0.02 ng/mL	[129]
Labeled		CAP	Fish	Au MNPs-Apt-SSB + ds-DNA Ab/EV-AuNPs-HRP	0.015 ng/mL	[120]
Labeled		CAP	Milk	3 HRP-mimicking DNAzymes: Fe-MIL-88 (MOFs)-Pt NPs-sDNA	0.03 pM (0.0097 pg/mL)	[130]
Labeled		CAP	Milk powder	Hemin/G-quadruplex DNAzyme-Au NPs	0.13 pg/mL	[131]
Label-free		CAP	Milk	AuNPs aggregation + Apt	0.03 nM (0.0097 ng/mL)	[132]
Label-free	SULFA	SDMX	Raw milk, honey, egg	LCs	10 ng/mL	[133]
Labeled (dc-ELAA)	TETRA	OTC	Chicken muscle, milk, honey	Aptamer + OTC-HRP	0.88 ng/mL	[134]
Labeled (ic-ELAA)		OTC	Milk	Aptamer-HRP	12.3 ng/mL	[135]
Labeled (dc-ELAA)		Tetracycline	Honey	Aptamer + TTC-HRP	0.0978 ng/mL	[136]
Labeled (ic-ELAA)		Tetracycline	Honey	Aptamer-HRP	9.6 × 10^−3^ ng/mL	[137]
Label-free		Tetracycline	Milk	Intrinsic peroxidase-like activity of AuNCs	46 nM (20.4 ng/mL)	[138]
Label-free		Tetracycline	Milk	CS-AuNPs aggregation+ Apt	39 ng/mL	[139]
Label-free		Tetracycline	Milk	AuNPs aggregation + Apt	266 pM (0.12 ng/mL)	[140]
Label-free		Tetracycline	Milk	AuNPs aggregation + Apt + PDADMAC	1 μM (444.4 ng/mL) (naked eyes), 45.8 nM (20.4 ng/mL) (detector)	[141]
Dual colorimetric (Label free) and fluorescence (Labeled)	AMGL	Streptomycin	Milk	AuNPs aggregation + Apt	73.1 nM (42.5 ng/mL) (colorimetric) and 47.6 nM (27.7 ng/mL) (fluorescence)	[142]
Dual colorimetric and fluorescenceLabel-free	BL	Ampicillin	Milk	AuNPs aggregation + FAM-Apt	10 ng/mL (colorimetric) and 2 ng/mL (fluorescence)	[143]

AMGL: aminoglycosides; Apt-Au-PV, gold nanoparticles (AuNPs) aptamer (Apt) co-immobilized on PV (PowerVision™); Apt-SiO_2_@Au-HRP, horseradish peroxidase on the core-shell SiO_2_@Au nanoparticles; AuNCs, gold nanoclusters; Au NPs-SSB, labeling SSB on gold magnetic particles; Apt-SiO_2_@Au-HRP, core-shell SiO_2_@Au nanoparticles; BL, beta-lactams; CAP, chloramphenicol; CS-AuNPs, cysteamine-stabilized gold nanoparticles; dc-ELAA, direct competitive enzyme-linked aptamer assay; ELAA, enzyme-linked aptamer assay; FAM-Apt, 5′-fluorescein amidite-modified aptamer; Fe-MIL-88-Pt-DNAzyme, MIL-88, platinum nanoparticles (Pt NPs), and single-strand DNAzyme (Hemin/G-quadruplex); HRP-AuNPs, horseradish peroxidase modified AuNPs; ic-ELAA, indirect competitive enzyme-linked aptamer assay; LCs, liquid crystals; MULTI, multiplex; NMOF-Pt-sDNA, signal labeled nano metal-organic frameworks-platinum nanoparticles; OTC, oxytetracycline; PDADMAC, poly(diallyldimethylammonium chloride); PHENI, phenicolated; SULFA, sulfonamides; TETRA, tetracyclines; TTC, tetracycline.

**Table 3:** Other optical aptasensors for the detection of antimicrobial residues in food products.

Detection Technique	Analyte	Food Product	Detection Principle	Limit of detection (LOD) (ng/mL or nM)	References
Chemiluminescence	Sulfamethazine	Milk	Luminol-H_2_O_2_ + SMZ-HRP + Apt	0.92 ng/mL	[148]
	Kanamycin	Milk	Luminol-H_2_O_2_ + cDNA-AuNCs + Apt-MBs	0.035 nM (0.02 ng/mL)	[149]
	OTC, TTC, Kanamycin	Milk	ABEI-H_2_O_2_-PIP + cDNA-AuNFs + Apt-MNPs	0.02, 0.02 and 0.002 ng/mL	[49]
	CAP	Milk	ABEI-H_2_O_2_-PIP + cDNA-AuNFs + Apt-MNPs	1 ng/mL	[40]
Luminescence	Kanamycin	Fish	SPLPt(II) + Apt	143 nM (83.3 ng/mL)	[150]
ECL	Kanamycin	Milk	Luminol-H_2_O_2_ + AgNPs-Apt (catalyser)	0.06 ng/mL	[151]
FQ-EWA	Kanamycin	Milk	Cy3-Apt + GO + AAP	26 nM (15.1 ng/mL)	[43]
SPR	Neomycin B	Buffer	Apt	5 nM (3.1 ng/mL)	[152]
SERS	Kanamycin	Milk	Au@AgNPs-cDNA + Cy3-Apt	0.90 pg/mL	[153]
	OTC	Milk	AuNPs-Apt + AuNPs-cDNA1 + AuNPs-cDNA2 + AuNPs-cDNA3	4.35 × 10^−3^ fg/mL	[154]
	OTC	Fishmeal	AuNPs (13 nm)-cDNA-AuNPs (80 nm) + Apt + 4-MBA	4.35 × 10^−3^ fg/mL	[155]
	Tetracycline	Milk	MCNCs-PMAA-MNs-Apt + Au/PATP/SiO_2_-cDNA	0.001 ng/mL	[156]
	CAP	Milk	Au@Ag NSs-cDNA-Cy5-Apt	0.19 pg/mL	[157]
RRSS	Tobramycin	Milk	AuNPs-Apt + CuSO_4_	0.19 nM (0.09 ng/mL)	[158]

AAP, anchor aptamer probe on fiber; ABEI, N-(4-aminobutyl)-N-ethylisoluminol; AgNPs, silver nanoparticles; Apt, aptamer; Au@Ag NSs, Au core-Ag shell nanostructures; AuNCs, gold nanoclusters; AuNFs, flower-like gold nanostructures; AuNPs, gold nanoparticles; cDNA, complementary DNA; Cy5, cyanine 5; ECL, electrochemiluminescence; FQ-EWA, evanescent wave aptasensors based on target binding facilitated fluorescence quenching; GO, graphene oxide; 4-MBA, 4-mercaptobenzoic acid; MCNCs-PMAA-MNs, magnetite colloid nanocrystal clusters-polymethacrylic acid magnetic nanospheres; MNPs, magnetic nanoparticles; OTC, oxytetracycline; PIP, P-iodophenol; PLNPs, persistent luminescence nanoparticles; RRSS, resonance Rayleigh scattering spectra; SERS, surface-enhanced Raman scattering; SPEET, surface plasmon enhanced energy transfer; SPLPt(II), square-planar luminescent platinum(II) complex; SPR, surface plasmon resonance.
